# Prolonged starvation deepens quiescence in Vasa2/Piwi1-expressing cells of a sea anemone

**DOI:** 10.1371/journal.pbio.3003525

**Published:** 2025-12-08

**Authors:** Eudald Pascual-Carreras, Kathrin Garschall, Patrick R. H. Steinmetz

**Affiliations:** Michael Sars Centre, University of Bergen, Thormøhlensgate, Bergen, Norway; University of California Los Angeles, UNITED STATES OF AMERICA

## Abstract

Animals with lifelong growth adjust their growth rates to nutrient availability, yet the underlying cellular and molecular mechanisms remain poorly understood. Here, we studied how food supply and TOR signaling regulate the cell cycle in a multipotent population of Vasa2-/Piwi1-expressing cells in the sea anemone *Nematostella vectensis*. We discovered that starvation induces a reversible G_1_/G_0_ cell cycle arrest in Vasa2+/Piwi1+ cells and that cell cycle re-entry upon refeeding is dependent on TOR signaling. In addition, the length of the refeeding stimulus after starvation determines the proportion of cells that re-enter S-phase. Remarkably, prolonged starvation delayed both refeeding-induced TOR signaling activation and S-phase re-entry, and led to a global decrease in the active histone mark H3K27ac in Vasa2+/Piwi1+ cells. This strongly suggests that *Nematostella* Vasa2+/Piwi1+ cells undergo starvation-controlled quiescence deepening, a phenomenon previously described only in unicellular eukaryotes and mammalian cell culture. The nutritional control of quiescence and cell proliferation may thus be a fundamental, evolutionarily conserved strategy underlying the environmental regulation of indeterminate growth in animals.

## Abstract 2

Animals with lifelong growth modulate cell proliferation in response to nutrient availability, but the underlying mechanisms are not fully understood. This study shows that starvation induces a reversible G1/G0 cell cycle arrest in Vasa2+/Piwi1+-expressing Nematostella vectensis cells and cell cycle re-entry upon refeeding is dependent on TOR signaling.

## Introduction

Animals with indeterminate body size retain the ability to regulate their growth rates throughout life in response to changing environmental conditions. Examples of such animals are found across diverse bilaterian (e.g., fish, crustaceans, annelids) and most non-bilaterian lineages, including sea anemones (e.g., *Nematostella vectensis*), corals, and sponges [1–4]. Lifelong growth and high body plasticity are therefore likely ancestral to all animals [2,5]. Some animals with indeterminate growth dramatically adjust their body size to environmental changes, shrinking during starvation and regrowing upon refeeding. Examples of whole-body shrinkage include marine iguanas [6], planarians [7], and many cnidarians such as sea anemones (e.g., *Nematostella vectensis*) [4], hydrozoans (e.g., *Hydra vulgaris*) [8,9], or jellyfish (e.g., *Pelagia noctiluca*) [10]. In contrast, most biomedical model organisms (i.e., mammals, nematodes, and flies) exhibit fixed, genetically pre-determined body sizes with growth uncoupled from feeding at maturity. In these animals, starvation depletes stored nutrients, causes tissue atrophy (e.g., in muscle), and arrests stem cell proliferation in germline or high-turnover tissues, such as the intestinal epithelium [11–13]. Limited data from animals with lifelong growth leave the cellular, molecular, and physiological links between nutrient availability and growth regulation poorly understood, especially beyond embryonic development, regeneration, or injury response.

On a cellular level, the nutritional regulation of the cell cycle is well studied in unicellular eukaryotes such as yeast [14–16], green algae (*Chlamydomonas reinhardtii*) [17], and mammalian cell cultures (e.g., fibroblasts) [18,19]. In these systems, nutrient depletion typically induces cellular quiescence, defined as a reversible cell cycle arrest that occurs mostly during the G_1_/G_0_ or, more rarely, the G_2_ phase of the cell cycle [17–20]. In vertebrates and flies, cellular quiescence is predominantly observed in adult stem cells (e.g., muscle, hematopoietic, neural, intestine). Their reactivation occurs upon regeneration, injury, or growth factor stimulation, but is rarely linked to dietary conditions. Notably, adult mammalian stem cells occasionally exit quiescence and divide spontaneously to support tissue renewal, enabling their identification by long-term label retention [21,22].

Nutritionally regulated quiescence has been identified in only few animal cells, such as larval neuroblasts in *Drosophila* [23,24], neural progenitors in *Xenopus* [25], and germline stem cells in *C. elegans* [26,27], as well as in the adult mouse brain [28]. The target of Rapamycin (TOR) signaling pathway, largely conserved among eukaryotes, controls nutritional reactivation of the cell cycle in some animals (e.g., *Drosophila*, *Xenopus*) [25,29,30] but not others (e.g., *C. elegans*) [27]. The predominance of non-nutritional cues in regulating animal stem cell quiescence and proliferation has led to speculations that following the evolution of multicellularity, quiescence became controlled by secreted growth factors rather than nutrients [31,32]. Since animals with indeterminate size depend on nutrient-regulated growth control, studying sea anemones may be key to understanding the significance and evolution of starvation as a trigger to induce cellular quiescence in animals.

Although cellular quiescence was discovered decades ago, its metabolic and genetic regulation has only recently begun to be elucidated [33–35]. In both animals and yeast, quiescence is an actively maintained state characterized by increased chromatin compaction, changes in histone modifications, and reduced metabolic, transcriptional, and translational activity [20,36–38]. Work in mammalian cell culture has introduced the concept of ‘quiescence depth’ to explain variations in the degree of quiescence, where cells in deeper quiescence require stronger stimuli, exhibit delayed cell cycle re-entry, and show greater transcriptomic shifts compared to those in shallow quiescence [18,19,33,35,39–42]. Prolonged deprivation of nutrients or growth factors can lead to loss of proliferative competence and/or to senescence in cultured cells [18,20,43]. Similar variations in ‘readiness’ to re-enter the cell cycle were found in adult mammalian stem cells, with increased TOR complex 1 activity marking the transition from G_0_ to a ‘G_alert_’ or a primed state [22]. Recent studies in fibroblast cell cultures indicate that quiescence depth is modulated by the duration of nutrient or growth factor deprivation and is regulated by autophagy, lysosomal activity, and the Retinoblastoma/E2F transcription factor network [18,42,43]. Whether the nutritional control of quiescence depth has organismal relevance, particularly in the context of body size plasticity and growth regulation, remains unclear.

**Fig 1:**
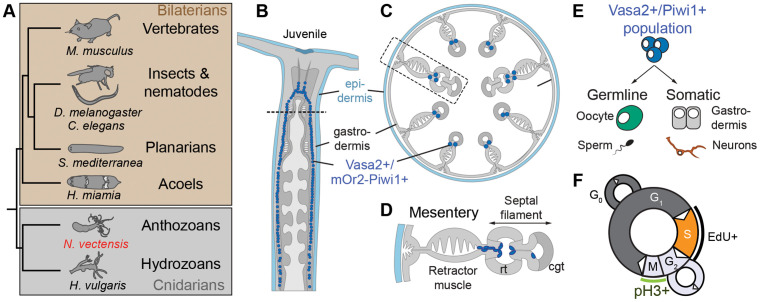
The phylogenetic position of *Nematostella* and localization of Vasa2 +/Piwi1+ cells within the juvenile polyp body plan. **(A)** Simplified phylogenetic tree highlighting the phylogenetic position of the sea anemone *Nematostella vectensis* and other animal taxa relevant for this study. All animal silhouettes are licensed under CC0,1.0 Universal Public domain and taken from https://www.phylopic.org. **(B–D)** Schematics showing the localization of Vasa2+/Piwi1+ cells in a juvenile polyp, depicted in longitudinal **(B)** or cross-section **(C, D)**. **(E)** Schematic representation of the multipotent, Vasa2+/Piwi1+ stem/progenitor cell population and a simplified summary of their germinal and somatic progeny. **(F)** Schematics of cell cycle phases, highlighting the incorporation of EdU during S-phase (black line) and the phosphorylation of Histone H3 (pH3+) during metaphase. Full species names in (A): *Caenorhabditis elegans*, *Drosophila melanogaster, Hofstenia miamia*, *Hydra vulgaris, Mus musculus*, *Nematostella vectensis*, and *Schmidtea mediterranea.* cgt: cnidoglandular tract. rt: reticular tract.

On a whole-body level, the relationship between feeding, cell cycle dynamics, and body size plasticity has been mainly studied in *Hydra* and planarians (e.g., *Schmidtea mediterranea*) (Fig 1A) [7–9,44–48]. In starved *Hydra*, interstitial and epithelial stem cells remain slowly proliferative, with extended S and G_2_ phases enabling rapid cell cycle acceleration upon refeeding [48–50]. Similarly, in planarians, pluripotent neoblasts continue dividing during starvation [46,51,52]. In both organisms, starvation-induced whole-body shrinkage results from increased cell loss relative to proliferation, without apparent quiescence of stem cells in G_1_ [44,46].

The sea anemone *Nematostella vectensis* has emerged as a valuable research organism for studying body plasticity in response to environmental changes, including nutrient availability and temperature [4,53–56]. In *Nematostella*, feeding is necessary for body growth from the primary polyp stage onwards, and for the addition of new tentacles in juveniles [57]. A recent study showed that in juvenile *Nematostella* polyps, feeding triggers body growth and cell proliferation, while starvation induces whole-body shrinkage [4]. Notably, this starvation-induced body shrinkage is fully reversible, highlighting that juvenile growth plasticity is part of the normal developmental repertoire of *Nematostella* to resist nutritional challenges [4]. However, the stem or progenitor cell populations driving this plasticity and their responses to feeding or starvation remain unexplored. We therefore studied a recently identified proliferative cell population in *Nematostella* that co-expresses conserved germline and multipotency genes (e.g., *Vasa2, Piwi1*) [58]. These Vasa2+/Piwi1+ cells constitute ~0.05% of all cells in a fed juvenile polyp. Their cell lineage has recently begun to be elucidated by studying the cytoplasmic inheritance of mOrange2 (mOr2) or GFP fluorophores in the transgenic Piwi1^P2A-GFP^ knock-in and promotor-driven *vasa2*::mOr2 [58] and *piwi1*::mOr2 lines [59]. These studies showed that Vasa2+/Piwi1+ cells contribute to both germline and somatic cells, including *soxB(2)+* neuronal progenitor cells, and represent a putative multipotent stem cell population, although their homogeneity remains to be investigated (Fig 1B–1E) [58,59]. Because fluorophores become degraded and diluted during growth and cell division, our current understanding of the Vasa2+/Piwi1+ lineage remains incomplete. Moreover, it is unclear if the recently described, broad expression of the germline/multipotency marker *nanos2* also encompasses Vasa2+/Piwi1+ cells [59].

Here, we study the response of Vasa2+/Piwi1+ cells to starvation and re-feeding in juveniles, revealing cellular and epigenetic hallmarks of deepening quiescence under prolonged starvation. We further demonstrate that their cell cycle re-entry upon refeeding depends on TOR signaling. Our findings suggest that the nutritional regulation of quiescence depth, previously characterized in unicellular organisms and cell cultures, may be an evolutionary conserved mechanism underlying animal growth plasticity.

## Results

### The cell cycle of Vasa2+/Piwi1+ cells responds dynamically to food availability

**Fig 2:**
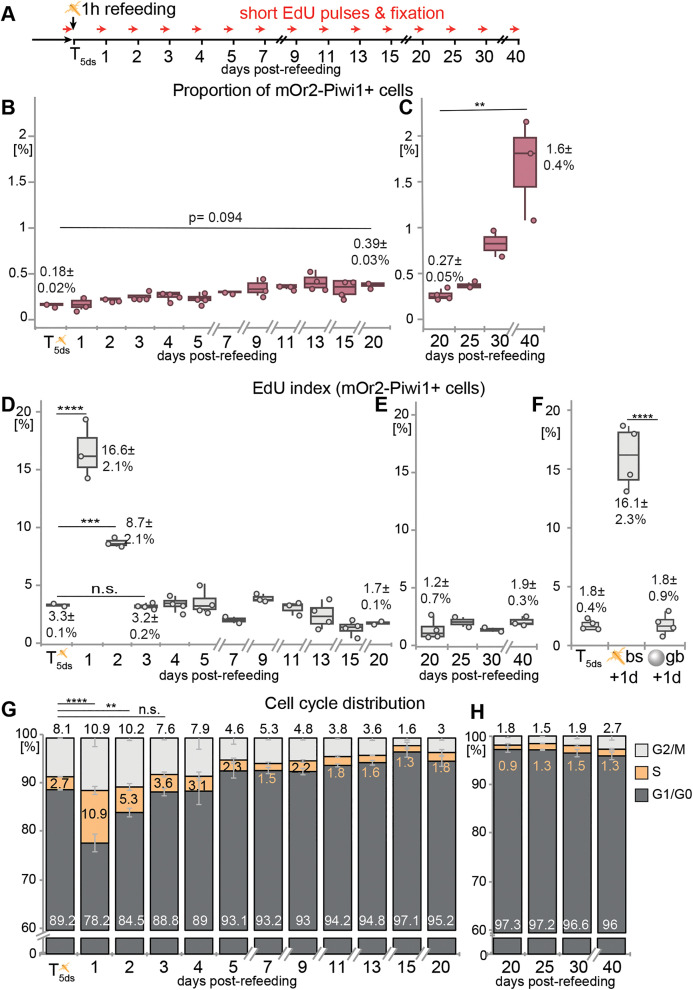
Feeding and starvation affect the proportion and cell cycle activity of Vasa2+/Piwi1+ cells. **(A)** Schematic illustrating the sampling and experimental procedure. Polyps were refed for 1 hour after 5 days starvation (T_5ds_), sampled at indicated days post-refeeding and measured by flow cytometry. 30-min-long EdU pulses were performed before fixation. **(B, C)** The proportion of mOr2-Piwi1+ cells increases between T_5ds_ and 20 days post-refeeding **(B)**, and between 20 and 40 days post-refeeding **(C)**. **(D, E, G, H)** Refeeding at T_5ds_ triggers a transient peak in cell proliferation as indicated by changes in the proportion of EdU+ cells (EdU index; **D, E**) and cell cycle phase distributions **(G, H)** among all Vasa2+/Piwi1+ cells. **(F)** Brine shrimps (‘bs’), but not BSA-coated glass beads (‘gb’) induce S-phase re-entry 24 hours after incubation at T_5ds_. See Data visualization for definition of box plots and bar plots. Values in B–F represent means ± standard deviations of respective timepoints with dots representing individual samples. Values in B–H represent means ± standard deviation. *n* = 2–4 biological replicates per condition, each replicate consisting of a pool of 15 animals. Significance levels after one-way ANOVA with Tukey’s HSD for pairwise comparisons are indicated for adjusted *p* values: **p < 0.01; ***p < 0.001; ****p < 0.0001. bs: brine shrimp; d: day(s); gb: glass beads; n.s.: non-significant. See S1 Table for mean values and statistical data and S1 Data for individual numerical values.

Cell proliferation in juvenile *Nematostella* polyps is tightly regulated by food supply. Feeding induces a transient burst of proliferation throughout the polyp, which subsides to low baseline levels within five days [4]. We investigated how the Vasa2+/Piwi1+ cell population responds to nutrient availability in juvenile polyps (see Materials and methods) before gametogenesis introduces a nutritional trade-off between somatic growth and sexual reproduction. The effect of feeding and starvation on cell proliferation at a whole-body level had been previously studied on the same stage [4]. To synchronize the feeding status and cell cycle across all polyps, we starved juveniles for five days before refeeding at T_5ds_. Changes in the relative abundance, proliferation and cell cycle phase distribution of Vasa2+/Piwi1+ cells were then quantified by flow cytometry during the subsequent 40 days after refeeding (Fig 2A). We dissociated juveniles of the transgenic Piwi1^mOr2^ knock-in line, which expresses high levels of mOr2-Piwi1 protein specifically in Vasa2+/Piwi1+ cells [58]. These cells represent a much smaller cell population than those labeled in the Piwi1^P2A-GFP^, *vasa2*::mOr2 [58] or *piwi1*::mOr2 lines [59], all of which include large numbers of progeny cells. We have then used flow cytometry (FC) to gate and analyze Piwi1^mOr2^ cells with high mOr2-Piwi1 intensities (see Materials and methods; S1A–S1L Fig). Using the same gating strategy on negative control samples (i.e., no primary antibody), we found that the false-positive detection rate lies below 0.003% (0 out of 31,679 cells; S1H and S1I Fig; S1B Table).

The proportion of Vasa2+/Piwi1+ cells remarkably increased by ~2.2-fold between T_5ds_ and 20 days of starvation, and by ~6.2-fold between 20 and 40 days of starvation (Fig 2B and 2C; S1A–S1C and S1F Table). To determine relative changes in proliferation rates, we combined flow cytometry with ‘snapshot’ 30-minute incubation pulses of 5-ethynyl-2′-deoxyuridine (EdU), a thymidine analogue that incorporates into replicating DNA and allows labeling cells progressing through S-phase during the incubation window (Figs 1F and S1A–S1K). In addition, we used DNA content measurements determined by flow cytometry to quantify cells in G_1_/G_0_ (2N DNA content), S (between 2N and 4N), or G_2_/M (4N) cell cycle phases (Figs 1F and S1A–S1J).) [4]. At T_5ds_, the time point when juveniles were refed after 5 days of starvation, only 3.3 ± 0.1% of Vasa2+/Piwi1+ cells incorporated EdU, indicating a low proliferation rate (Fig 2D). Twenty-four hours after refeeding, the relative proportion of EdU+ cells among all Vasa2+/Piwi1+ cells (i.e., EdU index) increased by ∼5-fold, in accordance with a 4-fold increase in S-phase cells as determined by DNA content (Fig 2G). Both values approximately halved by 48 h and returned to baseline within three days of refeeding (Fig 2D and 2G; S1A, S1B, S1D, and S1E Table). The decrease in G_1_/G_0_- and simultaneous minor increase in G_2_/M-phase cell proportions (Fig 2G) indicate that refeeding prompted cell cycle re-entry from G_1_/G_0_. The sharp, transient surge in the EdU index and proportion of S-phase cells in Vasa2+/Piwi1+ cells further suggests that S-phase re-entry occurred relatively synchronously. To differentiate between the effect of nutritional input and any mechanical or sensory cues during feeding, we tested whether ‘feeding’ of BSA-coated, ~150–210 µm-sized glass beads at T_5ds_ triggers cell proliferation 24 hours later. We found that bead uptake did not induce any significant changes in the EdU index among mOr2-Piwi1+ cells, or among all cells (Figs 2F and S1M–S1O; S1I and S1J Table), showing that nutrients are essential to induce cell cycle re-entry in juvenile polyps.

Between 3 and 40 days of starvation, the proportion of Vasa2+/Piwi1+ cells in S-phase remained low without showing any further S-phase peaks (EdU: 1.2%–3.2%; S: 0.9%–3.6%; Figs 2D, 2E, 2G, 2H, S1A, and S1B; S1D, S1E, S1G, and S1H Table). Altogether, these findings suggest that Vasa2+/Piwi1+ cells continue to proliferate at constantly low rates during prolonged starvation.

### Feeding duration determines proliferative competence

**Fig 3:**
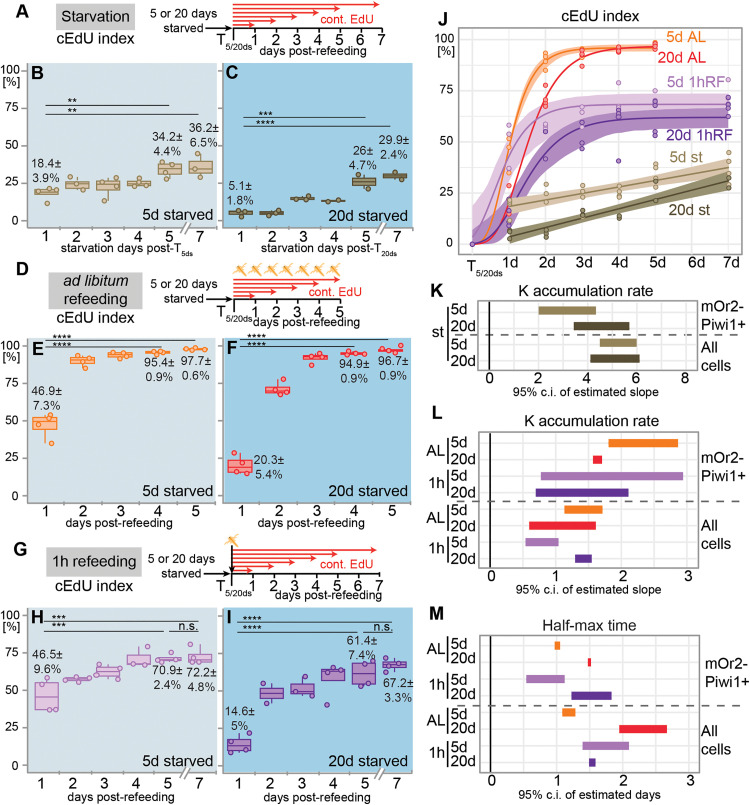
The effect of feeding and starvation on the proliferative competence and cell cycle re-entry dynamics of Vasa2+/Piwi1+ cells. **(A, D, G)** Schematics illustrating the sampling, feeding regimes, and EdU incubations. After 5 or 20 days of starvation (T_5ds_ or T_20ds_), polyps were either continuously starved **(A)**, refed *ad libitum* **(D)**, or refed with a single, 1-hour refeeding pulse **(G)** and incubated in EdU between 1 and 7 days. **(B–C, E–F, H–I)** Temporal changes in the cumulative EdU (cEdU) index under continued starvation **(B, C)**, *ad libitum* refeeding **(E, F)**, or following a single, 1-hour refeeding pulse **(H, I)** after T_5ds_ **(B, E, G)** or T_20ds_ **(C, F, I)**. Experiments were done using flow cytometry. **(J)** Dynamics of the cEdU index are best explained by linear growth models under continued starvation (st), or by Gompertz growth models after *ad libitum* (AL) or a 1-hour refeeding pulse (1hRF). Dots represent the same replicate sample values as in **(B, C, E, F, H, I)**. **(K, L)** Estimating the effect of starvation **(K)**, *ad libitum*, and 1-hour refeeding **(L)** on the accumulation rate of cEdU+ cells was done by comparing the 95% confidence intervals between 5 and 20 days of starvation. K rate in **(K)** derived from linear models **(J)**, and K rate in **(L)** derived from Gompertz growth models **(J)**. **(M)** Estimating the effect of *ad libitum* and 1-hour refeeding on the half-max time, when the cEdU index reaches 50% of its maximum, was done by comparing the 95% confidence intervals between the experiments started at T_5d_ and T_20d_. Half-max time derived from Gompertz growth models **(J)**. ‘All cells’ refers to all cell cycle-gated cells including mOr2-Piwi1+ cells (S2 Fig). n = 2–4 biological replicates per condition (15 individuals per replicate). Coloured lines in J represent the model curve or line for each condition with overlays depicting 95% confidence intervals. SeeData visualization for definition of box plots. Dots represent individual values. Index values represent means ± standard deviations of respective timepoints. Pairwise comparisons after one-way ANOVA were calculated using Tukey’s HSD and *p* values adjusted at significance codes: **p < 0.01; ***p < 0.001; ****p < 0.0001. d: day(s), n.s.: non-significant. See S2, S3, and S4 Tables for mean values and statistical data and S1 Data for individual numerical values.

While feeding induced a sharp but transient proliferation burst, it remained unclear whether all or only a subset of Vasa2+/Piwi1+ cells were responsive to the feeding stimulus. To investigate how the duration of starvation and the length of refeeding pulses impact their proliferative competence, we assessed cell cycle re-entry in Vasa2+/Piwi1+ cells following different starvation periods (Fig 3A, 3D, and 3G). We continuously provided EdU (cEdU) over 5–7 days to determine the cumulative EdU index (cEdU index), which reflects the proportion of cells that progressed through at least one S-phase since T_5ds_ or T_20ds_. In the continued absence of food, ~30%–36% of Vasa2+/Piwi1+ cells were labelled by cEdU over 7 days, regardless of the preceding starvation duration (Figs 3A–3C and S2A–S2J; S2A and S2L Table). This indicates that starvation duration has no major effect on the rate of spontaneous proliferations.

Over 5 days *ad libitum* (AL) refeeding, ~ 96%–97% of Vasa2+/Piwi1+ cells accumulated cEdU, independent of the prior starvation duration (Figs 3D–3F and 6A; S2D and S2K Table). Thus, almost all Vasa2+/Piwi1+ cells proliferated during 5 days of AL refeeding. In contrast, the cEdU index among all polyp body cells, which include terminally differentiated cells that cannot re-enter the cell cycle, is ~ 13%–25% lower (S3C and S3D Fig; S3D and S3K Table). Following a single, 1-hour refeeding pulse (Fig 3H and 3I), the cEdU index measured after 5 or 7 days was well above starvation baseline but significantly lower than during AL refeeding (Figs 3B, 3C, 3E, 3F, 3H, 3I, and 6A; S2A, S2D, S2G and S2K Table). Notably, starvation duration had no significant effect on the proportion of Vasa2+/Piwi1+ cells re-entering the cell cycle, but a single, 1-hour-long refeeding stimulus resulted in a lower proliferative competence compared to AL-refed polyps (Figs 3E, 3F, 3H, 3I, 6A, and S3C–S3F).

**Fig 4:**
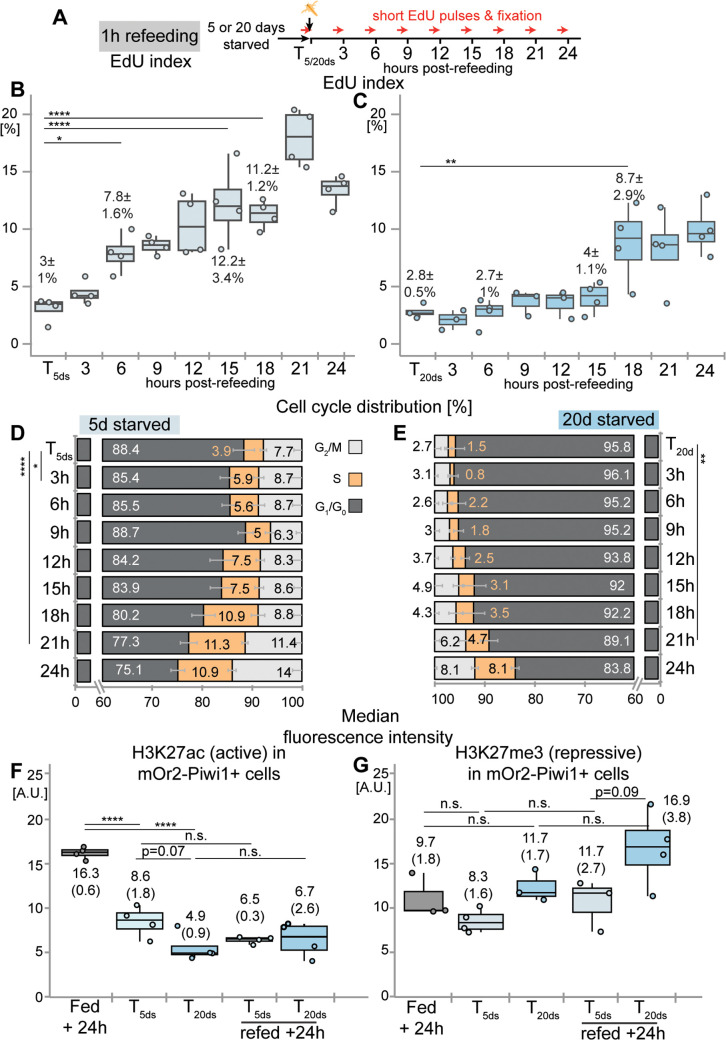
Prolonged starvation leads to a delay in cell cycle re-entry and decrease in H3K27ac in Vasa2+/Piwi1+ cells. **(A)** Schematic illustrating the sampling, feeding regimes, and EdU incubations. Polyps starved for 5 or 20 days (T_5ds_ or T_20ds_) were refed for 1 hour and sampled at indicated hours post-refeeding. Thirty min-long EdU pulses were performed for each sample before the fixation. **(B–E)** Flow cytometry-based quantification of the proportion of EdU+ cells (EdU index; **B, C**) and cell cycle phase distributions **(D, E)** over 24 hours after polyps were refed at T_5ds_ or T_20ds_. Following refeeding at T_5ds_ and T_20ds_, the earliest significant changes in the EdU index **(B, C)** and S-phase proportion **(D, E)** occur at 6 and 18 hours **(B, C)** or at 3 and 21 h **(D, E)** post-refeeding, highlighting the delayed cell cycle re-entry after 20 days of starvation. Note that the proportions of S and G_2_/M phase cells are higher at T_5ds_ than at T_20ds_ **(D, E)**. **(F, G)** Comparison of the median fluorescence intensity (MFI) of H3K27ac **(F)** and H3K27me3 **(G)** at 24 h following continuous feeding (Fed + 24 h), T_5ds_, T_20ds_, or at 24 h post-refeeding (T_5ds_/T_20ds_ refed + 24 h). During starvation (T_5ds_, T_20ds_), MFI levels of H3K27ac progressively decreased **(F)** while levels H3K27me3 did not change significantly **(G)**. For box plots and bar plot definitions, see Data visualization. Values in B-E represent means ± standard deviations and values in F and G represent the median (interquartile range) of respective timepoints with dots indicating individual samples. *n* = 2–4 biological replicates per condition, with 15 polyps per replicate. Significance levels after one-way ANOVA with Tukey’s HSD for pairwise comparisons are indicated for adjusted *p* values: *p < 0.05, **p < 0.01; ****p < 0.0001. d: day(s), n.s.: non-significant. See S5 and S6 Tables for mean values and statistical data and S1 Data for individual numerical values.

**Fig 5:**
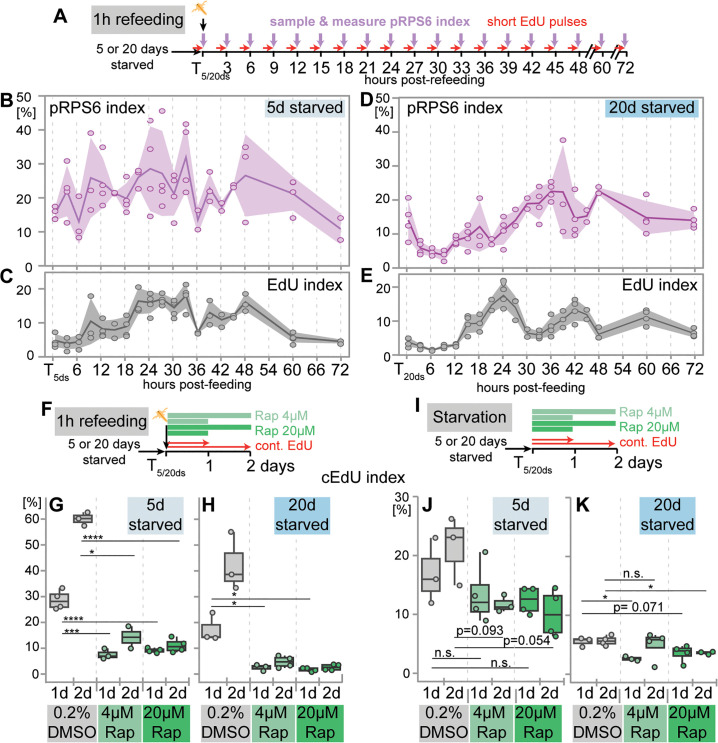
Dynamics and functional role of TOR signaling during cell cycle re-entry in Vasa2+/Piwi1+ cells after refeeding. **(A, F, I)** Schematics illustrating the feeding procedures, the TOR inhibitor Rapamycin (‘Rap’) and EdU incubation conditions, and sampling timepoints. After 5 or 20 days of starvation (T_5ds_ or T_20ds_), polyps were either refed by a single, 1-hour feeding pulse **(A, F)** or continuously starved **(I)**. EdU incubations were either for 30 min EdU **(A)** or continuously at indicated durations **(F, I)**. **(B–E)** Changes in the proportion of pRPS6+ (pRPS6 index; **B, D**) and EdU+ cells (EdU index; **C, E**) over 72 hours after refeeding at T_5ds_ **(B, C)** or T_20ds_ **(D, E)**. Experiments were done using flow cytometry. Note similarities in the pRPS6 and EdU index dynamics within the first 24 h after refeeding. Coloured lines indicate mean per condition and band overlays represent 95% confidence intervals. *n* = 2–4 biological replicates per condition, with 15 individuals per replicate. Dots represent individual values. **(G–K)** Rapamycin inhibits feeding-induced cell proliferation as shown by the suppression of the proportion of cumulative EdU+ cells (cEdU index) in Rapamycin-treated samples compared to DMSO-treated controls **(G)**. During continued starvation, Rapamycin has little impact on the cEdU index compared to DMSO controls **(J, K)**. For box plots, see Data visualization. Dots represent individual values. *n* = 2–4 biological replicates per condition, with 15 individuals per replicate. Significance levels for Student *t* test are indicated for *p* values: *p < 0.05, ***p < 0.001, ***p < 0.0001. d: day(s). See S7 and S8 Tables for mean values and statistical data and S1 Data for individual numerical values.

**Fig 6:**
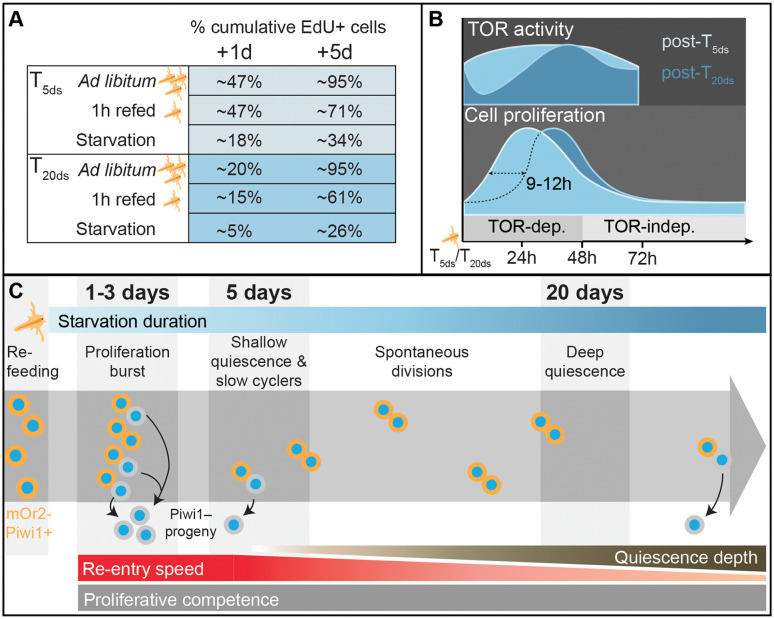
Schematic summaries of the nutritional regulation of Vasa2+/Piwi1+ cell proliferation and quiescence. **(A, B)** Schematic summaries depicting temporal differences in proliferation competence **(A)**, and in TOR activity, sensitivity and cell cycle re-entry **(B)** of Vasa2+/Piwi1+ cells between animals refed after T_5ds_ or T_20ds_. The proliferation competence is shown as approximate proportion of cumulative EdU+ cells among all Vasa2+/Piwi1+ cells. **(C)** Working hypothesis based on our results indicating that feeding triggers a burst of cell proliferation in Vasa2+/Piwi1+ cells, while starvation induces cellular quiescence. Prolonged starvation deepens quiescence, characterized by a delayed cell cycle re-entry and TOR signaling activity increase upon refeeding. After short-term starvation (5 days), a subset continues to divide slowly and asymmetrically. Under continued starvation, some cells spontaneously exit quiescence and divide symmetrically. Even after 20 days of starvation, there was no detectable decline in the competence of Vasa2+/Piwi1+ cells to re-enter the cell cycle upon refeeding. dep.: dependent; h: hours; indep.: independent.

### Feeding stimulus and starvation history affect the dynamics of cell cycle re-entry

We then asked how starvation history affects the dynamics of EdU+ cell accumulation by comparing the proportion of Vasa2+/Piwi1+cells accumulating EdU within the first day (1d-cEdU index) across feeding regimes and starvation durations. Notably, the 1d-cEdU index was consistently ~2–3 times higher at T_5ds_ (Fig 3B, 3E, and 3H) compared to T_20ds_ (Fig 3C, 3F, and 3I) throughout all feeding/starvation conditions (Fig 6A; S2A, S2D, S2G, and S2J Table). Remarkably, this difference between short- and long-term starved polyps is also found when analyzing all polyp cells of the 1h and AL refeeding samples, suggesting that this phenomenon is not restricted to Vasa2/Piwi1+ cells (S3A–S3F Fig; S3A, S3D , S3G and S3J Table). Notably, the 1d-cEdU index of Vasa2/Piwi1 + cells from polyps sharing the same starvation history (i.e., T_5ds_ or T_20ds_) were not significantly different between AL or 1-hour refeeding (compare 1d-cEdU index between Fig 3E and 3H or between Fig 3F and 3I; S2D, S2G, and S2J Table). These results suggest that starvation history but not feeding regime affects the proportion of Vasa2/Piwi1+ cells re-entering the cycle during the first 24 h after refeeding.

To determine whether these differences arise from changes in the rate or onset of proliferation, we estimated cEdU index dynamics by applying linear regression or growth models to the data. During continuous starvation, a single-phase linear regression model proved a good fit to the datapoints between 1 and 7 days of EdU incubation (Fig 3J and S4A Table; 5d: *R*^2^ = 0.6063; 20d: *R*^2^ = 0.8543). We observed that independent of the starvation history, the cEdU index linearly increased between 1 and 7 days of continuous starvation without apparent stagnation (T_5ds_[Δd7/1]: ~2-fold; T_20ds_[Δd7/1]: ~6-fold; Fig 3J and S4A Table). All Vasa2+/Piwi1+ cells may therefore eventually proliferate even under prolonged starvation. Extrapolation predicted that the cEdU index among Vasa2+/Piwi1+ cells would have reached 100% after 31.5 (T_5ds_) and 21.9 days (T_20ds_). Notably, the cEdU accumulation rate (K) was significantly lower (i.e., shallow slope) after 5 than 20 days of starvation, with minimal overlap in their 95% confidence intervals (Fig 3J and 3K; S4A Table). Interestingly, K_20d_ of Vasa2+/Piwi1+ cells resembled values from all polyp cells as their 95% confidence intervals largely overlapped (Figs 3K and S3G ; S4A Table). These findings suggest that EdU+ cell accumulation in Vasa2+/Piwi1+ cells is unusually slow after short starvation but normalizes following prolonged starvation to a level generally observed among all proliferating cells. Differences in accumulation rates among Vasa2+/Piwi1+ cells between short and long starvation may reflect a switch from an asymmetric (i.e., production of a Piwi1− progeny cell) to a symmetric cell division mode (i.e., division into two Piwi1+ cells; see Discussion).

After AL refeeding, a Gompertz Growth model [60,61] proved an excellent fit to describe the dynamics of measured cEdU indices (5d: *R*^2^ = 0.999; 20d: *R*^2^ = 0.998; Figs 3J, S4A–S4F, and S4M–S4R; S4B Table). From this model, we estimated the accumulation rate K (Figs 3L and S4Z) and the half-max time t50 (Fig 3M), which represents the time point at which the cEdU index reaches 50% of its maximum (S4Z Fig). Following AL refeeding, K_5d_ was significantly higher than K_20d_, with non-overlapping 95% confidence intervals (Fig 3L; S4C Table), while t50_20d_ was reduced by about half a day compared to t50_5d_ in Vasa2+/Piwi1+ cells (Fig 3M; S4C Table). Among all cells, AL refeeding after long starvation did not change cEdU accumulation rates but delayed t50 by about a full day (Figs 3L, 3M, and S3G; S4B and S4C Table). Together, these results indicate that prolonged starvation slows and delays EdU+ cell accumulation in Vasa2+/Piwi1+ cells upon AL refeeding.

To assess whether this pattern persisted following a 1-hour refeeding stimulus, we again fitted a Gompertz Growth model (5d *R*^2^ = 0.9685; 20d *R*^2^ = 0.9796; Figs 3J, S4G–S4L, and S4S–S4Y; S4B Table). While *K* values were not different, t50 was significantly delayed after 20 days of starvation, mirroring AL refeeding results (Fig 3L and 3M; S4C Table). Together, these findings suggest that starvation history affected the onset but not the rate of cEdU accumulation in Vasa2+/Piwi1+ cells under any refeeding regime.

### Starvation length affects onset of cell cycle re-entry

To assess whether lower K and higher t50 values after prolonged starvation reflect delays in cell cycle re-entry, we analyzed the ‘snapshot’ EdU index (i.e., 30 min EdU incubation) at high temporal resolution following a single 1-hour refeeding stimulus (Fig 4A). To control for potential circadian effects, we found that sampling in the morning or evening did not significantly affect EdU indices, the proportion of phospho-histone H3+ cells [62,63] or cell cycle distributions of Vasa2+/Piwi1+ cells from polyps starved for 5 or 20 days (S5A–S5C and S7A–S7K Figs; S5A and S5B Table).

Following T_5ds_, the EdU index increased significantly within 6 hours of refeeding, whereas after T_20ds_, a significant increase was only observed at 18 hours and onwards (Fig 4B and 4C; S5A, S5C, and S5F Table). This 12–15-hour delay in S-phase re-entry was further confirmed by flow cytometry-based DNA content analysis (Fig 4D and 4E; S5A and S5D–S5G Table). Although the EdU index between T_5ds_ and T_20ds_ was not significantly different (Fig 4B and 4C; S5A and S5B Table), the proportions of S and G_2_/M phase cells were markedly lower after prolonged starvation (Fig 4D and 4E; S5A and S5B Table). The proportion of S-phase cells measured by DNA content significantly increased already within 3 hours of re-feeding at T_5ds_, while after 20 days of starvation, a significant increase was detected only after 21 hours (Fig 4D and 4E; S5A and S5D–S5G Table). Together, these findings strongly suggest that prolonged starvation delays S-phase re-entry in Vasa2+/Piwi1+ cells (Fig 6B).

To determine if the delay in re-entry was linked to the reduced proportion of G_2_/M phase cells after 20 days starvation, we quantified the proportion of phosphorylated Histone H3 (pH3 index), a mitosis marker (Fig 1F) conserved between fungi, plants, and animals [64]. No significant changes in the pH3 index were observed in either starvation condition within the first 15 hours post-feeding (S5D–S5F Fig; S5A, S5E, and S5H Table), suggesting that cell cycle re-entry is not driven by G_2_/M-phase cells but rather by a relatively synchronous re-entry from G_1_/G_0_.

### Starvation leads to global changes in active but not repressive chromatin marks among Vasa2+/Piwi1+ cells

Previous studies showed that cellular quiescence in yeast and animals is associated with epigenetic changes in histone post-translational modifications (PTMs) that regulate chromatin accessibility and transcriptional activity [36,38,65]. Whether and how chromatin marks change during quiescence deepening remains unclear [38]. To gain first insights into the epigenetic changes occurring between proliferative and quiescent Vasa2+/Piwi1+ cells, we studied global changes in the acetylation or trimethylation of lysine 27 on histone H3 (i.e., H3K27ac or H3K27me3). In bilaterians and *Nematostella*, H3K27ac marks active enhancers and promotors, while H3K27me3 labels Polycomb repressor complex 2-mediated long-term repression of enhancers and promotors [66,67]. Using confocal imaging, we confirmed that immunofluorescence against both histone PTMs specifically label nuclei at different signal intensities within the same sample (S6A–S6L′ Fig). To determine changes in global H3K27ac and H3K27me3 levels in Vasa2+/Piwi1+ cells, we measured the median fluorescence intensity (MFI) of each histone PTM using flow cytometry analysis (S7L Fig). We observed that H3K27ac MFI levels among Piwi1-mOr2+ cells decreased significantly between 24 hours after continuous feeding and T_5ds_ (to ~52%) or T_20ds_ (to ~34%), respectively (Fig 4F and S6A Table). Between T_5ds_ and T_20ds_, H3K27ac MFI levels showed a decreasing trend, which, however, failed to pass the statistical significance threshold (*p* = 0.07; Fig 4F; S6A and S6B Table). Similarly, significant decreases in H3K27ac levels between fed, T_5ds_ and T_20ds_ were observed among all cells (S6M Fig; S6E and S6F Table). Together, our findings show a quantitative and progressive loss of H3K27ac during quiescence deepening in Vasa2+/Piwi1+ cells, and potentially other cell populations, which may indicate that starvation leads to a global decrease in active transcription. In contrast, levels of the repressive H3K27me3 mark did not change significantly among Vasa2+/Piwi1+ cells (or among all cells) between continuously fed or refed polyps and any starvation samples (T_5ds_ or T_20ds_; Figs 4G and S6G; S6C, S6D, S6G, and S6H Table). Our results thus do not indicate any major quantitative shifts in the repressive H3K27 trimethylation mark during starvation or refeeding.

### Starvation length affects feeding-dependent TOR signaling in cycling Vasa2+/Piwi1+ cells

To investigate the molecular mechanisms of delayed cell cycle re-entry after prolonged starvation, we examined whether TOR signaling activity correlates with these differences. As a readout of active TOR signaling, we used phosphorylation of the ribosomal protein S6 (pRPS6), which has previously been established in yeast [68], sea anemones [4,57,69] and bilaterians [70]. Using flow cytometry (S8A–S8K Figs), we quantified changes in the proportions of EdU+ (EdU index) and pRPS6+ cells (pRPS6 index) to assess TOR activity in relation to S-phase re-entry after short- and long-term starvation. To rule out circadian effects, we confirmed that EdU and pRPS6 index levels and the proportions of cell cycle phases did not significantly differ between Vasa2+/Piwi1+ cells from unfed T_5ds_- or T_20ds_-polyps sampled 9 hours apart (S9A–S9C Fig; S7A and S7B Table). Between T_5ds_ and T_20ds_, pRPS6 indices were not significantly different (S9A Fig; S7A and S7B Table).

Following 1-hour refeeding, the pRPS6 indices showed considerable variation at individual time points, but overall, their dynamics differed between short- and long-term starvation histories (Fig 5A, 5B, and 5D; S7C Table). After short starvation, pRPS6 indices increased within hours and remained high (20%–30%) up to 48 hours post-refeeding. In contrast, after 20 days of starvation, pRPS6 indices initially declined to <5% over 9 hours before reaching 20%–30% only at 36 h (Fig 5A, 5B, and 5D; S7C Table). The dynamics of the EdU index confirmed the delay in S-phase re-entry previously observed after T_20ds_, with a slightly earlier increase at 15 hours (Figs 5C, 5E, and 6B; S7C Table) compared to the 18-hour time point previously identified (Figs 4B, 4C, and 6B). The temporal changes in pRPS6 levels closely mirrored those of the EdU index. Pearson correlation analysis showed a positive correlation between pRPS6 and EdU indices over 72 hours after T_5ds_ (Fig 5B and 5C; S7C and S7D Table), but only during the first 30 hours after T_20ds_ (Fig 5D and 5E; S7C and S7E–S7G Table). These findings suggest that TOR signaling activity is closely associated with S-phase re-entry, particularly during the early phase after re-feeding (Fig 6B), raising the question if TOR acts in parallel or upstream of S-phase re-entry and whether TOR is predominantly active in proliferating cells.

Confocal imaging confirmed that pRPS6 protein is present during both S-phase (S9D–S9H Fig; co-labeling with EdU) and M-phase (S9E–S9K Fig; co-labeling of cell with metaphase plate). To determine whether pRPS6+  cells were enriched during specific cell cycle phases, we compared cell cycle distributions between pRPS6+ and pRPS6− fractions (S10A and S10B Fig; S7H Table). Calculating the log_2_FC of their ratio (pRPS6+/pRPS6−), we found that S- and G_2_/M-phase cells were over-represented (log_2_FC > 0) across nearly all timepoints, while G_1_/G_0_ cells were underrepresented (S10C and S10D Fig; S7I Table). These results indicate that proliferative Vasa2+/Piwi1+ cells predominantly exhibit active TOR signaling, regardless of starvation duration.

### TOR signaling is required for feeding-dependent cell proliferation in Vasa2+/Piwi1+ cells

To test whether TOR signaling is functionally required for refeeding-induced cell cycle re-entry, we treated polyps with two independent TORC1 inhibitors while refeeding: the allosteric inhibitor Rapamycin (‘Rap’) and the ATP-competitive inhibitor AZD-8055 (‘AZD’). Western Blot analysis confirmed that both inhibitors strongly suppressed pRPS6 levels in whole juvenile polyps at two concentrations, with Rapamycin being more effective overall at the studied concentrations (S11A–S11D Fig; S1 Raw Images; S8A–S8C Table).

EdU incorporation assays showed that both TOR inhibitors significantly reduced the EdU index and S-phase re-entry in Vasa2+/Piwi1+ cells after refeeding, irrespective of starvation length (S1A–S1K and S11E–S11J Fig; S8D, S8E, S8G, and S8H Table). Flow cytometry analysis further revealed that TOR inhibition reduced S-phase and G_2_/M-phase cell proportions while increasing G_1_/G_0_ proportions (S11K and S11L Fig; S8D, S8F, S8G, and S8I Table). Notably, Rap and AZD had a stronger effect on the EdU index (S11G–S11J Fig) than on the S-phase proportion determined by DNA content (S11K and S11L Fig). To test whether Rapamycin leads to a G_1_/S-phase arrest, or continued proliferation in a subset of cells, we assessed the cEdU index over 24 and 48 hours of continuous EdU incubation and Rapamycin incubation after refeeding (Fig 5F). We observed a marked reduction in the cEdU index, with a stronger effect after long starvation (Figs 5G, 5H, and S2A—S2J; S8J and S8K Table). Notably, the cEdU index at 1- and 2-days post-refeeding (Fig 5G and 5H; S8J Table) remained higher than the corresponding EdU index, regardless of starvation length (S11G and S11H Fig; S8D Table). This shows that even at high Rapamycin concentrations, a subset of Vasa2+/Piwi1+ cells (5d: ~7%–14%; 20d: 2%–5%) continue to enter S-phase within 1 and 2 days after refeeding, speaking against an arrest in G_1_ or the G_1_/S transition (Fig 5G and 5H; S8J Table). We also explored whether baseline proliferation of Vasa2+/Piwi1+ cells under continuous starvation is dependent on TOR signaling (Fig 5I). Following 5 or 20 days of starvation, we found that 24 h- or 48 h-cEdU levels resembled DMSO controls, indicating that baseline proliferation is largely Rapamycin-insensitive (Figs 5J, 5K, and 6B; S8L and S8M Table). Our findings indicate that cell cycle re-entry in Vasa2+/Piwi1+ cells is dependent on TOR complex 1 signaling after refeeding but not during starvation.

## Discussion

In the sea anemone *Nematostella vectensis*, body size and growth rates are tightly linked to nutrient availability, as is characteristic for animals with lifelong growth. Starvation leads to considerable cell losses and body shrinkage, while refeeding triggers cell proliferation and growth [4]. To understand how food availability regulates growth at the cellular level, we studied the nutritional regulation of Vasa2+/Piwi1+ stem/progenitor cells [58]. Their proportion increased by ~9.4-fold over 40 days of starvation, despite a >50% reduction in polyp size and total cell numbers during the same time period [4]. It is currently unknown whether the composition of cell types changes during starvation-induced shrinkage, except for the specific population of GLW+ neurons that scales with body shrinkage [53]. Whether the accumulation of Vasa2+/Piwi1+ cells results from a protection from cell loss or an expansion by continued proliferation will need further investigation.

Cell proliferation among Vasa2+/Piwi1+ cells was highly dependent on food availability. Refeeding after 5 days starvation induced a proliferation surge within 24 hours, indicated by a rapid increase in the EdU index and relative proportion of S- and G_2_/M phases. This burst was followed by a decline in the EdU index and S-phase proportion, with a complementary increase of G_1_/G_0_-phase cells within three days. These dynamics suggest that refeeding triggers a relatively synchronous re-entry into S-phase from a quiescent G_1_/G_0_ state (Fig 6C). Beyond 3 days of starvation, the proportion of S-phase/EdU+ cells remained stable at approximately 1%–3.5%, indicating spontaneous rather than synchronized cell divisions. This suggests that daily feeding induces a food-dependent circadian synchronicity in cell cycle progression, which—as we have shown experimentally—disappears during starvation, similar to previous observations in *Hydra* stem cells [50]. Together, our observations indicate that starvation and refeeding induce quiescence and cell cycle re-entry, respectively, in Vasa2+/Piwi1+ cells (Fig 6B and 6C).

While the EdU index among Vasa2+/Piwi1+ cells remained stable beyond 3 days of starvation, changes in cEdU dynamics and cell cycle phase distributions suggest altered cell cycle lengths and division patterns between 5 and 20 days of starvation. Notably, the proportion of cells accumulating cEdU over 7 days increased between T_5ds_ and T_20ds_, aligning with the rate observed among all cells. A previous study showed that under fed conditions, some of the progeny cells from Vasa2+/Piwi1+ cells are depleted of mOr2-Piwi1 protein, likely due to asymmetric cell division [58]. In our flow cytometry gating, their low mOr2-Piwi1 fluorescence levels lead to their exclusion from the Vasa2+/Piwi1+ cell pool, and from contributing to the cEdU+ index. The shift towards increased cEdU values between T_5ds_ and T_20ds_ may thus indicate that fewer EdU+ cells are lost from the Vasa2+/Piwi1+ cell pool at T_20ds_ due to a shift from predominantly asymmetric division at T_5ds_ to symmetric divisions under prolonged starvation (Fig 6C).

Between 5 and 20 days of starvation, both the cEdU index and G_2_/M proportion declined with a corresponding increase in G_1_/G_0_ cells. In apparent contradiction, the proportion of S-phase cells, as determined by the ‘snapshot’ EdU index, remained stable beyond 3 days of starvation. This discrepancy likely reflects a faster cell cycle progression at 5 days compared to 20 days, allowing more cells to pass through S-phase within 24 hours after short starvation. It also indicates that the subset of slowly proliferating Vasa2+/Piwi1+ cells decreases overall between 5 and 20 days of starvation. Under prolonged starvation, proliferative activity is persistently low but not restricted to a small subset of cells. Together, our observations indicate that quiescent Vasa2+/Piwi1+ cells spontaneously exit quiescence at low rates to potentially support tissue renewal (Fig 6C), as seen in mammalian intestinal or hematopoietic stem cells [21,22].

A defining hallmark of quiescent cells is their ability to re-enter the cell cycle in response to a specific stimulus. After 5 or 20 days of starvation, approximately 90%–97% of Vasa2+/Piwi1+ cells resided in G_1_/G_0_. Within five days of *ad libitum* refeeding, almost all cells (~96%–97%) re-entered the cell cycle from G_1_/G_0_ (Fig 6A and 6C). The remaining 3%–4% cells may be terminally differentiated, senescent, or may need additional stimuli (e.g., injury, growth factors) to exit quiescence.

A comparison between feeding regimes revealed that a single 1-hour refeeding pulse induced a proliferative response equivalent to 24 hours of *ad libitum* refeeding. However, polyps refed *ad libitum* exhibited an increased rate of EdU+ cell accumulation over the following days (Fig 6A). This suggests that feeding frequency, rather than the strength of the initial feeding stimulus, determines the proliferative competence of Vasa2+/Piwi1+.

In contrast to yeast and mammalian cell culture systems, where prolonged stimulus deprivation deepens quiescence and reduces the proportion of cells able to re-enter the cell cycle, starvation in *Nematostella* did not reduce the competence of Vasa2+/Piwi1+ to re-enter the cell cycle [18,39,42,71]. Even after 20 days of starvation, nearly all Vasa2+/Piwi1+ cells kept their full proliferative potential. Remarkably, however, refeeding after 20 days of starvation significantly delayed the onset of TOR signaling activation (i.e., RPS6 phosphorylation) and S-phase re-entry by 12–15 hours compared to refeeding after 5 days of starvation (Fig 6B). This delay explains the differences in the 1-day-cEdU indices found after *ad libitum* and 1-hour refeeding at T_5ds_ and T_20ds_. Altogether, we conclude that deepening quiescence in *Nematostella* Vasa2+/Piwi1+ cells is marked by delays in S-phase re-entry and TOR signaling activation upon refeeding (Fig 6B).

In bilaterians, stem cell quiescence is accompanied by changes in histone PTMs, such as H3K27 acetylation or trimethylation, which mark promotors and enhancers of genes that are actively transcribed (H3K27Ac) or repressed (H3K27me3) [36,38,65–67]. Depending on the model system and context, chromatin marks (e.g., H3K27me3) or global genome accessibility can increase or decrease during quiescence [38,72]. In *Nematostella* Vasa2+/Piwi1+ cells, we found a progressive loss of H3K27ac, indicating a potential decrease in the global number of active enhancers or promotors [66,67]. However, we found no significant global changes in H3K27me3, a more permanent marker for long-term repression [67], during prolonged starvation.

Also, levels of H3K27 acetylation do not change significantly within the first 24 hours of refeeding after any starvation duration, indicating that cell cycle re-entry does not require a global increase in re-acetylation of H3K27, and that other epigenetic or physiological processes may underlie the cell cycle re-entry delays observed at prolonged starvation. Future work, including a larger set of chromatin marks combined with an analysis of genome-wide chromatin accessibility changes, is necessary to reveal the potentially complex epigenetic remodeling occurring during quiescence and cell cycle re-entry in *Nematostella* Vasa2+/Piwi1+.

The simultaneous increase in RPS6 phosphorylation and S-phase re-entry raised the question if TOR signaling activation is required for or acts in parallel to quiescence exit. Inhibition of TOR complex 1 demonstrated that TOR signaling is necessary for feeding-induced cell cycle re-entry after short and long starvation. However, the proliferation of a subset of Vasa2+/Piwi1+ cells remains Rapamycin-insensitive after continuous short and long starvation, suggesting that spontaneous Vasa2+/Piwi1+ cell proliferation during starvation is TOR signaling-independent (Fig 6B).

In sea anemones, *Hydra* and planarians, starvation and refeeding trigger body shrinkage and regrowth through cell proliferation or cell loss, respectively [4,8,9,46,73]. Similar to *Hydra* interstitial stem cells and planarian neoblasts, *Nematostella* Vasa2+/Piwi1+ cells exhibits some key stem cell features, including a high nucleus-to-cytoplasm ratio and the expression of conserved germline multipotency program genes, such as *piwi* or *vasa* genes [58,74]. However, *Nematostella* Vasa2+/Piwi1+ cells are much scarcer than planarian neoblast (~30%–40% of all cells) [75] or *Hydra* interstitial stem cells (~15%–20% of all cells) [73]. Also, unlike neoblasts and stem cells in *Hydra* or *Clytia, Nematostella* Vasa2+/Piwi1+ accumulate during starvation and exhibit low baseline proliferation rates during long-term starvation [46,73,75,76]. Neoblasts and *Hydra* stem cells, in contrast, maintain or even increase mitotic activity during prolonged starvation [8,44,46,48,50–52,77]. In *Hydra*, the proportion of epithelial stem cells entering S-phase within 48 hours is ~8-fold higher than found in *Nematostella* Vasa2+/Piwi1+ cells after 20 days of starvation (~40% versus ~ 5%) [44]. *Hydra* stem cells also exhibit an extremely short or absent G_1_/G_0_ phase, while their G_2_ phase extends without arrest during starvation [8,44,50,77]. Together, these differences further highlight the tight nutritional control of quiescence and cell cycle re-entry in *Nematostella* Vasa2+/Piwi1+ cells, resembling the ancestral, nutrient-responsive cell cycle dynamics in yeast or mammalian fibroblast cells [20,34].

Quiescence depth following nutrient withdrawal has so far only been investigated in unicellular eukaryotes and mammalian cell culture. Our findings suggest that the nutritional control of proliferation and quiescence depth may have persisted in some animals, such as sea anemones, to maintain lifelong growth plasticity. It remains to be determined whether the molecular mechanisms regulating quiescence depth in cultured mammalian cells, such as the Retinoblastoma/E2F protein network [42] or lysosomal activity [18], are conserved in sea anemones. Our work helps understanding the mechanisms behind environmentally controlled body plasticity and lays the foundation for exploring the metabolic, transcriptomic and epigenetic changes during the reversible transition between proliferation and quiescence.

## Materials and methods

### *Nematostella* culture

*Nematostella vectensis* wildtype and transgenic polyps all derived from an original culture of CH6 females and CH2 males [78]. The genotype of transgenic polyps consisted of heterozygotes that resulted from a cross between homozygous *piwi1*^*mOr2*^ or *piwi1*^*P2A-GFP*^ knock-in lines [58] and wild-type animals from the original stock.

Adult females and males were maintained in *Nematostella* medium (NM) at a salinity of 16‰, a temperature of 18°C and under dark conditions. Adults were fed fresh *Artemia nauplii* 5 times per week, with a daily, partial exchange of water and a full cleaning of the culture boxes once a month. Spawning was induced approximately every three weeks by 12-hour light exposure combined with a temperature shift from 18 to 25°C [78,79]. Embryos were raised at 25°C and fed with mashed *Artemia nauplii* during the first week after reaching the polyp stage (∼10 days post-fertilization), followed by feeding with live *Artemia*. Sex could not be determined in juveniles therefore all data shown included both males and females. All experiments were performed on juvenile polyps of ∼5 mm in length just prior to the appearance of the 2nd pair of mesenteries.

### Feeding procedures

Prior to the experimental start, polyps were starved for 5 (T_5ds_) or 20 days (T_20ds_), representing short or long starvation periods. At these time points, they were either kept starved or refed *ad libitum* or for 1 hour. During starvation periods, dishes were cleaned using cotton swabs and NM was replaced two times per week. After 1-hour feedings, polyps were transferred to a new petri dish and NM was changed twice per week. Under *ad libitum* feeding conditions, polyps were fed twice in excess and transferred to clean dishes daily, containing fresh *Artemia*.

To facilitate sampling, the 1-hour refeeding time point was delayed by 3, 6, and 9 hours, respectively (21/45-, 18/42-, and 15/39-hours post-feeding samples). To assess potential circadian rhythmicity in cell proliferation dynamics, the T_5ds_ +9 hours or T_20ds_ +9 hours timepoints were sampled for each starvation condition.

For differentiating between the effect of nutritional input and any mechanical or sensory cues during feeding, we compared the EdU index of mOr2-Piwi1+ and all cells from animals that took up glass beads to *Artemia* fed polyps. To encourage uptake, acid-washed glass beads sized between 150 and 212 microns (G1145, Sigma-Aldrich) were soaked for 10 mins in NM/5% BSA and transferred to 5-day-starved animals. The EdU index was determined by 90 min EdU incorporation 23 hours after a 1 h feeding pulse with brine shrimps or 24 h incubation with BSA-soaked glass beads. 24 h after feeding started, *Artemia*-fed polyps had expelled all food debris while polyps still retained glass beads within their body column.

### EdU labeling

*Nematostella* polyps were relaxed with 0.1 M MgCl_2_ in NM before being transferred to NM containing 100 µM EdU (Invitrogen), 2% DMSO and 0.1 M MgCl_2_, or 2% DMSO (controls). Animals were incubated at room temperature (RT) for a ‘snapshot’ 30-min. EdU pulse and washed with NM containing 0.1 M MgCl_2_ prior to the sampling time point (so from 2h30 until 3 h for the ‘3 h’ sample). For the bead feeding experiment, the EdU pulse was performed for 90 min. In continuous EdU pulse experiments, either in starved or *ad libitum* conditions, animals were incubated at 25°C without MgCl_2_, and the EdU or DMSO solution was replaced every 24 hours. In this context, T_5ds_ and T_20ds_ represent the time point before the onset of EdU exposure or the application of any nutritional condition. Flow cytometry analysis was performed after Trypsin/formaldehyde-based cell dissociation and fixation (see below). For microscopy sample preparation, polyps were fixed overnight in 3.7% formaldehyde/1× PBS, washed three times with 1× PBS/0.5% Triton X-100, and stored in 100% methanol (see below).

### Trypsin/formaldehyde-based cell dissociation and fixation

Juveniles were dissociated in pools of 10–15 polyps following the published protocol [4], with the resulting cell suspensions treated as biological replicates. Briefly, polyps were relaxed in 0.1 M MgCl_2_, washed with Ca^2+^- and Mg^2+^-free *Nematostella* medium (CMF/NM) followed by CMF/NM containing 0.195% ethylenediaminetetraacetic acid (CMF/NM+E). Then, the polyps were incubated for 5 min at 37°C in preheated CMF/NM+E containing 0.25% Trypsin (w/v). Animals were dissociated by pipetting and cold CMF/NM containing 1% BSA and 2.5% of Fetal Bovine Serum was added to stop trypsinization. Cells were pelleted at 800*g* for 5 min at 4°C and resuspended in 1% BSA/1× PBS (w/v). After filtering through a pre-wetted 50 µm CellTrics strainer (Sysmex) the cell suspension was fixed with 3.7% formaldehyde for 30 min at RT in the dark. Finally, the cells were washed twice with 1% BSA/PBS by spinning at 800 g for 5 min at 4°C. The final cell pellet was resuspended in 90% Methanol/0.1%BSA/0.1×PBS in H_2_O and stored at −20°C. Before use, cell suspensions were rehydrated by washing twice with cold 1%BSA/PBS (800*g* for 5 min at 4°C) and stained for flow cytometry.

### S-phase detection and immunofluorescence on cell suspensions

After rehydration (see above), cells were permeabilized with 0.2% Triton X-100 in PBS for 15 min at RT and washed with 1× PBS. The cell pellet was resuspended in 50 µl freshly prepared Click-iT reaction cocktail containing Alexa488 (ThermoFisher, C10337) or Alexa647 fluorophore azide (ThermoFisher, C10340) for 30 min at RT in the dark, following manufacturer’s instructions. Cells were washed twice with 0.2% Triton X-100 in PBS (800*g* for 5 min at 4°C). Cell suspensions were stained by immunofluorescence as previously described [58]. In short, cells were blocked in 1× PBS/10% DMSO/5% NGS/0.2% Triton X-100 for 30 min at RT and incubated in primary antibody solution 1× PBS/0.1% DMSO/5% NGS/0.2% Triton X-100 overnight at 4°C in the dark. The primary antibodies used were rabbit anti-DsRed 1:500 (Takara Bio Clontech 632496), mouse anti-mCherry 1:100 or 1:500 (Takara/AH Diagnostics 632543-CLO), rabbit anti-p-RPS6 (Ser235/236) 1:500 (Cell Signaling Technology, 4858), rabbit anti-pH3 (Ser10) 1:500 (Cell Signaling Technology, 9701), rabbit anti-H3K27me3 1:500 (Diagenode, C15410068) and rabbit anti-H3K27ac 1:500 (Abcam, Ab4729). After two washes with 1x PBS/0.2% Triton X-100 (800*g* for 5 min at 4°C), cell suspensions were incubated with the secondary antibody in 1× PBS/0.1% DMSO/5% NGS/0.2% Triton X-100 for 30 min at RT in the dark. The secondary antibodies used were goat-anti-rabbit-Alexa488/568 1:500 (LifeTech, A-11008, A11011) and goat-anti-mouse-Alexa568 1:500 (LifeTech, A11004). Negative controls were stained with the secondary antibody only. Finally, cells were washed twice with 1× PBS/0.2% Triton X-100 (800*g* for 5 min at 4°C) and resuspended in 1% BSA/PBS containing 1 µg/ml FXCycle Violet (ThermoFisher, F10347) for DNA staining. Cells were stored at 4°C and analyzed by flow cytometry within 24 hours without further washing.

### Flow cytometry analysis

Flow cytometry was performed on a LSRFortessa cell analyzer (BD Life Sciences) equipped with 407, 488, 561, and 640 nm lasers. Cell cycle phases were distinguished using FXCycle violet DNA dye, detected with the BF450/50 filter. Incorporated EdU was coupled with Alexa647 fluorophore azide and detected using the BF670/14 filter. The mOr-Piwi1 protein was detected with antibodies against either dsRed or mCherry coupled with Alexa568 and analyzed with the BF610/20 filter. phosphorylated Histone 3, phosphorylated RPS6, and the post-translational histone modifications H3K27me3 and H3K27ac were detected using specific antibodies: anti-pH3 and anti-pRPS6, and anti-H3K27me3 and anti-H3K27ac. All antibodies were coupled with Alexa488 and measured with the BF530/30 filter. The resulting data were analyzed using FlowJoV10.9 (BD Life Sciences). Graphical representations of all gating strategies are shown in S1A–S1K, S2A–S2J, S7A–S7L, and S8A–S8K Tables.

For gating cells of interest, debris was excluded based on size and granularity using the FSC-A/SSC-A gate, followed by FSC-A/FSC-H to exclude doublets and FSC-A/SSC-W to remove high-complexity events. DNA dye intensity was then gated by area over height to refine event selection. A histogram of DNA dye intensity (area, linear scale) was used to identify characteristic peaks corresponding to 2N and 4N DNA content. These pre-selected events constituted the pool of cells used for downstream analyses such as cell cycle composition (subfractions based on DNA signal intensity), the fractions of EdU+, pRPS6+, pH3+, and mOr2-Piwi1+ cells, which were determined by gating fluorescence relative to negative controls, and the MFI. As previously described, we distinguished ‘low’ and ‘high’ mOr2 signal cells in the mOr2-Piwi1 population based on intensity and cell abundance. Throughout the manuscript, we referred to [mOr2-Piwi1]_high_ cells as mOr2-Piwi1+ or Vasa2+/Piwi1+ cells. The same gates were applied to analyze cell cycle composition and the fraction of EdU+, pRPS6+, and pH3+ cells and MFI within the population of mOr2-Piwi1+ cells. In the long-term EdU pulse, cell cycle composition was assessed based on DNA signal intensity without distinguishing S and G_2_/M populations.

### S-phase detection and immunofluorescence on whole-mount tissues

Polyps were relaxed using 0.1 M MgCl_2_, fixed in 3.7% Formaldehyde NM for 1 hour at RT and the physa removed with a scalpel in a petri dish. The remaining polyp was washed thoroughly in 1× PBS/0.2% Tween20, followed by dehydration in a series of methanol washes (20–50–100% methanol in 1× PBS/0.2% Tween20) and in several 100% methanol washes until all pigment was washed out. Samples were stored in 100% methanol at −20°C. When needed, tissues were progressively rehydrated in 1xPBS/0.2% Triton X-100. In S-phase labeling, incorporated EdU was ‘clicked’ to a fluorophore azide using the Click-iT EdU Cell Proliferation Kit for Imaging (Invitrogen, C10337), following the manufacturer’s protocol. After 30 min Click-it staining reaction, tissue pieces were washed with 1×PBS/0.2% Triton X-100. For immunofluorescence staining, tissue pieces were blocked in 1× PBS/10% DMSO/5% normal goat serum (NGS)/0.2% Triton X-100 for 2 hours at RT. Primary antibody incubation was performed in 0.1% DMSO/5% NGS/0.2% Triton X-100 at 4°C using the following antibodies: FluoTag-X4 anti-GFP 1:250 (NanoTag Biotechnologies, N0304) overnight and rabbit anti-p-RPS6 Ser235/236 1:200 (Cell Signaling Technology, 4858) over 3 nights. After washing with 1× PBS/0.2% Triton X-100, tissue was blocked in 1× PBS/5% NGS/0.2% Triton X-100 for 30 min at RT. Nuclear staining with Hoechst33342 (ThermoFisher, H3570) and secondary antibody incubation were performed in 1× PBS/5% NGS/0.2% Triton X-100 overnight at 4°C. The secondary antibody used was goat-anti-rabbit-Alexa568 (LifeTech A11011, A21244). Finally, tissue pieces were washed thoroughly in 1×PBS/0.2% Triton X-100, mounted on slides (Electron Microscopy Sciences, 63418-11) in 80% glycerol and sealed under a coverslip (Menzel-Gläser 18 × 18 mm) with clear nail polish.

### Confocal imaging

Immunofluorescence whole-mount tissue pieces were imaged on an Olympus FLUOVIEW FV3000 confocal microscope (standard PMT detectors) with 40× oil-immersion lens objective). The maximum projections stacks were processed, cropped, and adjusted for levels and color balance with ImageJ/Fiji [80,81].

### Drug treatment

A previously published Rapamycin incubation protocol for *Nematostella* [4,57] was adapted as follows: a 10 mM Rapamycin (Sigma-Aldrich, R8781) stock solution in 100% DMSO was diluted in NM to a final concentration of 4 μM or 20 μM. For AZD-8055, the protocol was adapted from a previously published protocol for *Exaiptasia* [69]: a 10 mM AZD-8055 (Nordic Bioste, HY-10422) diluted in 100% DMSO was diluted in NM to a final concentration of 0.1 and 1 μM. For all drug treatments, polyps were placed in drug solution for 2 hours before feeding. Fresh *Artemia nauplii* were added for 1 hour while polyps remained exposed to the drug. Afterwards, polyps were transferred into a fresh drug solution. Incubations occurred at 25°C in the dark, with solutions replaced daily. Rapamycin, AZD-8055 or 0.2% DMSO (control) treatments were conducted over 1 or 2 days. When combining Rapamycin treatment with a continuous EdU pulse, the same protocol was followed. After the feeding pulse, polyps were transferred into a solution containing both the drug and EdU.

### Western blotting

Protein was extracted from a pool of 50 juvenile polyps. After relaxation using 0.1 M MgCl_2_, polyps were transferred to homogenization tubes (M-tubes, Miltenyi Biotec, 130-093-236) containing RIPA buffer (150 mM NaCl, 50 mM Tris pH 8.0, 1%NP40, 0.5%DOC, 0.1% SDS) supplemented with cOmplete EDTA-free Protease Inhibitor Cocktail (Roche, 4693159001) and PhosphoStop inhibitor EDTA-free (Roche/Merck, 4906837001). Samples were incubated on ice for 30 min with intermittent mechanical disruption using the ‘protein_01_01′ program on the gentleMACS tissue homogenizer (Miltenyi Biotec, 130-093-235). The resulting homogenate was pelleted at 14,000*g* for 15 min at 4°C, and the supernatant was transferred to a new tube and stored at −80°C. Protein concentration was quantified using the Bradford Assay Kit (Thermo Fisher Scientific, 23246) following the manufacturer’s protocol. For SDS-PAGE, 20 µg of protein was mixed 4:1 with 4× Laemmli sample buffer (0.1 M Tris-HCl pH 6.8, 2%SDS, 20% Glycerol, 4% β-mercaptoethanol, 0.02% Bromophenol blue) and boiled for 5 min before loading. Proteins were resolved on 7.5% Mini-PROTEAN TGX precast gels (Bio-Rad, 4568096) in running buffer (25 mM Tris-HCl, 192 mM Glycine, 0.1% SDS) at 100 V for ∼90 min. The 10–250 kDa PageRuler Plus pre-stained protein ladder (Thermo Fisher Scientific, 26619) was used as a standard. Proteins were transferred to PVDF membranes using Trans-Blot Turbo Mini 0.2 µm PVDF Transfer Pack (Bio-Rad, 1704156) on a Trans-Blot Turbo transfer system (Bio-Rad) with the ‘mixed molecular weight’ program. Membranes were washed with 1× PBS/0.1% Tween (PBT) several times and blocked with 5% milk powder in PBT (MPBT) at RT for 1 h. Blots were cut at the 35 kDa band as a reference and incubated overnight at 4°C with the following primary antibodies in MPBT: rabbit anti-p-RPS6 (Ser235/236) (1:5,000; Cell Signaling Technology, 4858) and rabbit anti-Actin (1:2000; Sigma-Aldrich, A5060). Membranes were washed several times in PBT and incubated in goat anti-rabbit-HRP (1:10,000; Abcam, Ab9705) secondary antibody in MPBT at RT for 1 h. After further washes with TBT (20 mM TrisHCl, 150 mM NaCl, 0.1% Tween pH 7.6), signals were detected using Clarify ECL substrate (Bio-Rad, 1705060) and imaged with a ChemiDoc XRS+ (Bio-Rad). Quantifications were performed with ImageJ software by measuring pixel intensity per band and subtracting the background intensity. The pRPS6 protein signal was normalized to the Actin signal. Membranes are shown in S1 Raw Images.

### Data visualization

Data visualization and analysis were performed using Excel and R v4.2.2 statistical analysis software (https://www.r-project.org/, R Core Team, 2021) with the packages ggplot2 (v3.5.1) [82], dplyr (v1.1.4) [83], stats (v4.2.2, R Core Team 2022), pracma (v2.4.4) [84], minpack.lm (v1.2.4) [85], bbmle (v0.25.1) [86], and MASS (v3.58.1) [87].

Bar plots show the proportion of cell cycle phases, with the standard deviation of the lowest phase shown at phase intersections. Box plots show proportion of mOr2-Piwi1+ cells, EdU index, cEdU index, MFI, pRPS6 index, pH3 index, and western blot quantifications. The central line shows the median, while the bounds represent the 25th and 75th percentiles. Whiskers extend to the maxima within 1.5× the interquartile range above the upper quartile and to the minima within 1.5× the interquartile range below the lower quartile. Dots represent single replicate data points. Scatter plots show EdU index and pRPS6 index over time, each data point represents a biological replicate. The line represents the mean and the overlay represents the 95% confidence intervals. In model plots, dots represent biological replicates for each time point, lines show predicted values and shaded areas indicate 95% confidence intervals.

### Statistics

Statistical analysis included one-way ANOVA to estimate the effects of assay parameters (factors) followed by Tukey’s honest significant difference (HSD) test to evaluate pairwise differences between experimental groups. For parametric data comparison, a two-tailed Student *t* test (*α* = 0.05) was used. Correlations between p-RPS6 and EdU+ signal were estimated using Pearson correlation combined with Kolmogorov–Smirnov test, assessing normal Gaussian distribution. Values for the cEdU index under starvation condition was used as an input into linear regression model. Values for the cEdU index under *ad libitum* and 1-hour feeding conditions were used as input into Gompertz, Logisitic and Richards growth models.

Gompertz mode equation


$$P(t)=Pmax\cdot e^{-e^{-K(t-t\text{0})}}$$

Logisitic model equation


$$P(t)=\frac{Pmax}{\text{1}+e^{-K(t-t\text{0})}}$$

Richards model equation


$$P(t)=Pmax\cdot \left(\text{1}+e^{-K(t-t\text{0})-\frac{\text{1}}{m}}\right)$$

Akaike Information Criterion corrected (AICc) and coefficient of determination (*R*^2^) were calculated to support model selection. In sigmoidal growth models, steepness of the slope (*K*) and earlier half-max times (t50) obtained from Gompertz model indicate a faster response. Flow cytometry summary data and statistical analysis are provided in S1–S8 Tables and individual numerical values in S1 Data.

### Declaration of generative AI and AI-assisted technologies in the writing process

During the preparation of this work, the authors used ChatGPT (OpenAI, https://chat.openai.com) to draft code for data visualization and analysis, and for editing and polishing of the manuscript. After using this tool, the authors reviewed and edited the content as needed and take full responsibility for the content of the publication.

## Supporting information

S1 Fig Gating strategy of experiment using 30 min EdU pulses experiments in mOr2-Piwi1 juvenile polyps. **(A–C)** Debris was excluded based on size and granularity in the FSC-A/SSC-A gate (A), with sub-gates based on FSC-A/FSC-H (B) and FSC-A/SSC-W (C) to remove potential cell doublets and high-complexity events. **(D, E)** Then particles were gated based on DNA dye intensity in width-over-area plots, and a histogram of DNA dye intensity (area, linear scale) was created to visualize characteristic DNA peaks corresponding to cells between 2N and 4N. **(F, G)** A threshold for EdU+ cells was determined based on the fluorescence signal of DMSO controls within the 2N–4N pool. Cells above this threshold were considered as EdU+. **(H, I)** Similarly, a threshold for mOr2-Piwi1+ cells was drawn based on the fluorescence signal of negative controls (no primary antibody) within the 2N–4N pool, identifying small and bright cells as mOr2-Piwi1+. **(J, K)** Predefined cell cycle phases and EdU+ cells were then analyzed within this pool of cells. **(L)** Hierarchical logic used to define cell cycle phases, followed by quantification of mOr2-Piwi1+ cells and calculation of the EdU, cEdU, pH3, and pRPS6 indices, and the median fluorescent intensity of H3K27ac and H3K27me3 within mOr2-Piwi1+ cells. The parameters used for gating and analysis are specified at each step. **(M, N, O)** Incubation with brine shrimps (‘bs’; M, N), but not BSA-coated glass beads (‘gb’; M, O) induces S-phase re-entry 24 hours after incubation at T_5ds_. Representative stereomicroscopy images of juvenile polyps fed with brine shrimps (N) or BSA-coated glass beads (O) after approx. 1 h of incubation. Note that glass beads were taken up and expanded the body cavity similarly to brine shrimps. Scale bar: 0.5 mm. See Data visualization for definition of box plots and bar plots. Values in M represent means ± standard deviations with dots representing individual samples. *n* = 4 biological replicates per condition, each replicate consisting of a pool of 15 animals. Significance levels after one-way ANOVA with Tukey’s HSD for pairwise comparisons are indicated for adjusted *p* values: ****p < 0.0001. See S1 Table for mean values and statistical data and S1 Data for individual numerical values.
(TIF)

S2 Fig Gating strategy of experiments using continuous EdU incubation in mOr2-Piwi1 juvenile polyps. **(A–C)** Debris was excluded based on size and granularity in the FSCA-/SSC-A gate (A), with sub-gates based in FSC-A/FSC-H (B), and FSC-A/SSC-W (C) to remove potential cell doublets and high-complexity events. **(D, E)** Then, particles were gated based on DNA dye intensity in width-over-area plots, and a histogram of DNA dye intensity (area, linear scale) was created to visualize characteristic peaks corresponding to cells between 2N and 4N. We observed that long-term incorporation of EdU interfered with the DNA stain fluorescence and prevented a clear identification of 2N–4N cells. Therefore, we used a broader range of DNA intensity to define the parental gate of EdU+ populations. **(F, G)** A threshold for EdU+ cells was determined based on the fluorescence signal of DMSO controls within the pool. **(H, I)** Similarly, a threshold for mOr2-Piwi1+ cells was drawn based on the fluorescence signal of negative controls (no primary antibody), identifying small and bright cells as mOr2-Piwi1+. **(J)** The same gates were applied to analyze cell cycle composition and the fraction of EdU+ cells within this pool of cells.
(TIF)

S3 Fig The effect of feeding and starvation on the proliferative competence and onset of cell cycle re-entry in all cell cycle-gated cells. **(A–F)** Temporal changes among all cells in the cumulative EdU (cEdU) index under continued starvation (A, B), *ad libitum* refeeding (C, D), or following a single, 1-hour refeeding pulse (E, F) after 5 (T_5ds_, A, C, E) or 20 days (T_20ds_, B, D, F) of starvation. See Fig 1 for schematic of experimental setups. Experiments were done using flow cytometry. **(G)** Dynamics of the cEdU index (A–F) are best explained by linear growth models under continued starvation (st), or by Gompertz growth models after *ad libitum* (AL) or a 1-hour refeeding pulse (1hRF). Dots represent same replicate sample values as in (A–F). *n* = 2–4 biological replicates per condition (15 individuals per replicate). Coloured lines in G represent the model curve or line for each condition with overlays depicting 95% confidence intervals. See Data visualization for definition of box plots. Dots represent individual values. Index values represent means ± standard deviations of respective timepoints. Pairwise comparisons after one-way ANOVA were calculated using Tukey’s HSD and *p* values adjusted at significance codes: ****p < 0.0001. d: day(s), n.s.: non-significant. See S3 and S4 Tables for mean values and statistical data and S1 Data for individual numerical values.
(TIF)

S4 Fig Model selection to estimate the dynamics of EdU+ cell accumulation (cEdU) during *ad libitum* and 1-hour feeding conditions. **(A–Y)** Comparison of Gompertz (A, D, G, J, M, P, S, W), Logistic (B, E, H, K, N, Q, T, X), and Richards (C, F, I, L, O, R, V, Y) growth models for mOr2-Piwi1+ (A–L) and all cell cycle-gated cells (M–Y) during *ad libitum* (A–F, M–R) and 1-hour refeeding conditions (G–L, S–Y). The best fitting model for each condition was chosen based on the highest coefficient of determination (*R*^2^) and lowest Akaike Information Criterion corrected (AICc). Overall conditions, the Gompertz growth model performed best (red values). *n* = 2–4 biological replicates per condition (15 individuals per replicate). Black lines represent the growth model curve for each condition. Dots represent individual values. The Gompertz equation is displayed. **(Z)** The Gompertz growth model assumes a maximum value and exponential decay as the population approaches this maximum. The growth rate constant (*K*) controls how quickly the curve transitions and indicates the speed at which the predicted maximum (*P*max) is reached. The half-max time (*t*50) indicates the time point where the function reaches 50% of the *P*max. d: day(s). See S4 Table for mean values and statistical data and S1 Data for individual numerical values.
(TIF)

S5 Fig Proliferation rates of Vasa2+/Piwi1+ were independent on the daytime of the sampling, and mitotic rates were not significantly different upon refeeding of 5- or 20-day-starved polyps. **(A–C)** The cell cycle phase distribution (A), proportion of EdU+ cells (EdU index; B), and proportion of pH3+ cells (pH3 index; C) of Vasa2+/Piwi1+ cells from 5 or 20 days starved polyps (T_5ds_ or T_20ds_) sampled 9 hours apart showed no significant difference. **(D)** Schematic illustrating the sampling, feeding regimes, and EdU incubations. Polyps starved for 5 or 20 days (T_5ds_ or T_20ds_) were refed once for 1 hour and sampled at indicated hours post-refeeding. **(E, F)** Quantification of the pH3 index over 24 hours after refeeding in polyps starved for 5 or 20 days showed no significant difference between T_5ds_ or T_20ds_ and 15-h post-refeeding regardless of the starvation length. All experiments were done using flow cytometry. For box plots and bar plot definitions, see Data visualization. Dots in (B, C, F, G) represent individual values. Values in (A, E, F) represent means ± standard deviations at respective timepoints with dots representing individual samples. *n* = 3–4 biological replicates per condition, with 15 polyps per replicate. Pairwise comparisons after one-way ANOVA were calculated using Tukey’s HSD and *p* values adjusted at significance codes: *p < 0.05. n.s.: non-significant. See S5 Table for mean values and statistical data and S1 Data for individual numerical values.
(TIF)

S6 Fig Changes in the active H3K27ac and repressive H3K27me3 histone marks among Vasa2+/Piwi1+ and all cells in fed, starved, and refed juvenile polyps. **(A–L′)** Projections of confocal imaging stacks of cell clumps dissociated from juvenile polyps and immunolabeled against H3K27ac (A–F′) and H3K27me3 epitopes (G–L′). Both histone marks colocalized with nuclei labeled by Hoechst33342 DNA dye (A′–L′). Scale bar in A′–L′: 10 µm. **(M, N)** Comparison of the median fluorescent intensity (MFI) of H3K27ac (M) and H3K27me3 (N) at 24 h after continuous feeding (Fed + 24 h), at starvation (T_5ds_, T_20ds_) or at 24-h post-refeeding (T_5ds_/T_20ds_ refed + 24 h). Between fed, T_5ds_ and T_20ds_ timepoints, MFI levels of H3K27ac progressively and significantly decreased while levels H3K27me3 (M) did not change significantly (N). Starved polyps were refed for 1 hour. For box plots and bar plot definitions, see Data visualization. Values in M and N represent median and interquartile range (IQR) of respective timepoints with dots indicating individual samples. *n* = 2–4 biological replicates per condition, with 15 polyps per replicate. Significance levels after one-way ANOVA with Tukey’s HSD for pairwise comparisons are indicated for adjusted *p* values: *p < 0.05, **p < 0.01; ****p < 0.0001. d: day(s), n.s.: non-significant. See S6 Table for mean values and statistical data and S1 Data for individual numerical values.
(TIF)

S7 Fig Gating strategy of experiment using 30 min EdU pulses and pH3 detection in mOr2-Piwi1 juvenile polyps. **(A)** Debris was excluded, and cells were gated based on DNA dye intensity as described above. **(B, C)** For EdU+ cells, a threshold was drawn above the fluorescence signal of DMSO controls within the 2N–4N pool. **(D, E)** For pH3+ cells, a threshold was drawn based on the fluorescence signal of negative controls (no primary antibody) within the 2N–4N pool, identifying G2/M-phase cells as expected. **(F, G)** Similarly, for mOr2-Piwi1+ cells, a threshold was drawn based on the fluorescence signal of negative controls within the 2N–4N pool, identifying a small population of bright cells. **(H–J)** The same gates were applied to analyze cell cycle phases and the proportion of pH3+ and EdU+ cells within this pool of cells. **(K)** Comparison between the use of mCherry and dsRed antibodies for immunolabeling mOr2-Piwi1 cells. mOr2-Piwi1 was detected using both an mCherry antibody coupled with Alexa568 and a dsRed antibody coupled with Alexa488. Debris was excluded, and cells were gated based on DNA-dye intensity as explained above. For mOr2-Piwi1+ cells, thresholds were drawn based on the fluorescence signal of negative controls (no primary antibody) within the 2N–4N pool. A linear correlation between the fluorescent signals was confirmed. **(L)** Histogram comparing the fluorescent intensity distribution of a negative control and a sample stained for histone markers H3K27ac or H3K27me3. The median fluorescent intensity (MFI) for each population is indicated by a dashed line.
(TIF)

S8 Fig Gating strategy in 30-min EdU and pRPS6 experiments on mOr2-Piwi1+ cells from juvenile polyps. **(A)** Debris was excluded and gated cells based on DNA-dye intensity as explained above. **(B, C)** For analyzing EdU+ cells, a threshold was drawn above the fluorescence signal of DMSO controls within the 2N–4N pool of cells. **(D, E)** For pRPS6+ and pRPS6− cells, thresholds were drawn based on the fluorescence signal of negative controls (no primary antibody) within 2N–4N pools of cells. **(F, G)** For mOr2-Piwi1+ cells, a threshold was drawn based on the fluorescence signal of negative controls (no primary antibody) within 2N–4N pools of cells. **(H–K)** The same gates were applied to analyze cell cycle phases and the proportion of pRPS6+ and EdU+ cells within this pool of cells.
(TIF)

S9 Fig pRPS6 and proliferation rates of starved Vasa2+/Piwi1+ cells were independent on the daytime of refeeding, and phospho-RPS6 was found in S- and M-phase Vasa2+/Piwi1+ cells. **(A–C)** The proportion of phospho-ribosomal protein S6-positive cells (pRPS6 index; A), EdU+ cells (EdU index; B), and cell cycle phase distribution (C) of Vasa2+/Piwi1+ cells sampled 9 h apart at T5ds or T20ds showed no significant differences. Experiments were done using flow cytometry. For box plots and bar plot definitions, see Data visualization. Dots in (A, B) represent individual values. Values in (C) represent means. *n* = 2–4 biological replicates per condition, with 15 polyps per replicate. **(D–K)** Confocal imaging stacks of gastrodermal tissue from Piwi1P2A-GFP juvenile polyps. (D, E) Overview of mesenteries at midbody level of whole-mount polyps stained by immunofluorescence against pRPS6 sampled at 21 h or 24 h post-refeeding (hpf) after 5 days of starvation. Side views with oral side oriented downwards. **(F–H)** Single cells co-labeled by EdU, pRPS6, and Piwi1-(P2A-GFP)(white arrowheads). **(I–K)** A single metaphase cell co-labeled by pRPS6 and Piwi1-(P2A-GFP)(white arrow). EdU pulse labeling started 30 min before fixation. Gray: Hoechst DNA dye. Scale bar: 100 µm (D, E) and 10 µm (F–K). n.s.: non-significant. See S7 Table for mean values and statistical data, S1 Data for individual numerical values.
(TIF)

S10 Fig Overrepresentation of S and G_2_/M cells within the phospho-RPS6+ fraction of Vasa2+/Piwi1+  cells. **(A, B)** Flow cytometry-based cell cycle phase distributions of pRPS6+ and pRPS6− cells over 72 hours after refeeding at T_5ds_ (A) or T_20ds_ (B). Polyps were refed for 1 hour and sampled at indicated time points. For bar plot definitions, see Data visualization. Values in (A, B) represent means. *n* = 2–4 biological replicates per condition, with 15 polyps per replicate. **(C, D)** Log_2_FC of the ratio of the cell cycle fractions between pRPS6+ and pRPS6− cells. Note that regardless of the starvation duration, the pRPS6+ cells are overrepresented (Log_2_FC > 0) in the S and G_2_/M fractions upon refeeding. Dots represent individual samples. Coloured lines indicate mean values for each cell cycle phase and band overlays represent 95% confidence intervals. *n* = 2–4 biological replicates per condition, with 15 polyps per replicate. See S7 Table for mean values and statistical data and S1 Data for individual numerical values.
(TIF)

S11 Fig The TOR inhibitors Rapamycin and AZD-8055 strongly reduce RPS6 phosphorylation and cell proliferation in Vasa2+/Piwi1+ cells. **(A, B)** Western blots depict protein levels of phosphorylated ribosomal protein S6 (pRPS6) Actin (as control) after refeeding and incubating for 1 or 2 days with 0.2% DMSO, 4 µM or 20µM Rapamycin (‘Rap’, A) or 0.1 µM or 1µM AZD-8055 (‘AZD’, B). **(C, D)** Intensity measures of pRPS6 bands relative to the Actin control protein show that Rapamycin (C) and AZD-8055 (D) led to a decrease of phosphorylated RPS6 levels. *n* = 3–5 technical replicates from one biological replicate with pools of 50 Rapamycin-, AZD-8055- or 0,2% DMSO-treated polyps. **(E, F)** Schematics illustrating the feeding procedure, incubation conditions of EdU and the TOR inhibitors Rapamycin (‘Rap’, E) or AZD-8055 (‘AZD’, F), and sampling timepoints. After 5 or 20 days of starvation (T_5ds_ or T_20ds_), polyps were refed for 1 hour. **(G–L)** Effect of Rapamycin (G, H, K) and AZD-8055 (I, J, L) treatment on the proportion of EdU (EdU index, G–J) and cell cycle phase distribution (K, L) after a 1 h-feeding pulse at T_5ds_ or T_20ds_. Compared to 0.2% DMSO-treated controls, Rap (G, H) and AZD (I, J) treatment leads to a reduced EdU index regardless of starvation history, concentration, or incubation time. Rap (K) and AZD (L) reduce the fractions of S- and G_2_/M-phase cells. For box plots and bar plot definitions, see Data visualization. Dots represent individual values. *n* = 2–4 biological replicates per condition, with 15 individuals per replicate. Significance levels for Student *t* test are indicated for adjusted *p* values: *p < 0.05, ***p < 0.001, ***p < 0.0001. d: day(s), n.s.: non-significant. See S8 Table for mean values and statistical data, S1 Data for individual numerical values and S1 Raw Images for raw images of Western Blots.
(TIF)

S1 Table Details of statistical analyses applied related to Figs 2 and S1. **S1A Table.** Flow cytometer analysis after 30 min of EdU pulse—cell cycle phases defined as per DNA content. **S1B Table.** Flow cytometer analysis after 30 min of EdU pulse—cell cycle phases defined as per DNA content—extended starvation. **S1C Table.** ANOVA for the effect of **feeding and starvation day** on the fraction of **mOr2-Piwi1 high** and pairwise comparisons between days (Tukey’s HSD). **S1D Table.** ANOVA for the effect of **feeding and starvation day** on the **EdU index** and pairwise comparisons between days (Tukey’s HSD). **S1E Table.** ANOVA for the effect of **feeding and starvation day** on the fraction of **S-phase cells** and pairwise comparisons between days (Tukey’s HSD). **S1F Table.** ANOVA for the effect of **starvation day** on the fraction of **mOr2-Piwi1 high** and pairwise comparisons between days (Tukey’s HSD). **S1G Table.** ANOVA for the effect of **starvation day** on the **EdU index** and pairwise comparisons between days (Tukey’s HSD). **S1H Table.** ANOVA for the effect of **starvation day** on the fraction of **S-phase cells** and pairwise comparisons between days (Tukey’s HSD). **S1I Table**. Flow cytometer analysis after 90 min of EdU pulse—cell cycle phases defined as per DNA content—glass beads. **S1J Table.** ANOVA for the effect of **nutritional input** on the **EdU index** and pairwise comparisons between days (Tukey’s HSD).
(XLSX)

S2 Table Details of statistical analyses applied related to Fig 3. **S2A Table.** Flow cytometer analysis after continuous EdU pulse during starvation—cell cycle phases defined as per DNA content in mOr2-Piwi1 cells. **S2B Table. T**_**5ds**_—ANOVA for the effect of **starvation day** on the **cEdU index** and pairwise comparisons between days (Tukey’s HSD) —**mOr2-Piwi1 cells**. **S2C Table. T**_**20ds**_—ANOVA for the effect of **starvation day** on the **cEdU index** and pairwise comparisons between days (Tukey’s HSD)—**mOr2-Piwi1 cells**. **S2D Table.** Flow cytometer analysis after continuous EdU pulse during *ad libitum*—cell cycle phases defined as per DNA content in mOr2-Piwi1 cells. **S2E Table. T**_**5ds**_—ANOVA for the effect of ***ad libitum* day** on the **cEdU index** and pairwise comparisons between days (Tukey's HSD)—**mOr2-Piwi1 cells**. **S2F Table. T**_**20ds**_—ANOVA for the effect of ***ad libitum* day** on the **cEdU index** and pairwise comparisons between days (Tukey’s HSD) —**mOr2-Piwi1 cells**. **S2G Table.** Flow cytometer analysis after continuous EdU pulse after 1 h Refed—cell cycle phases defined as per DNA content in mOr2-Piwi1 cells. **S2H Table. T**_**5ds**_—ANOVA for the effect of **1 h Refed day** on the **cEdU index** and pairwise comparisons between days (Tukey’s HSD)—**mOr2-Piwi1 cells**. **S2I Table. T**_**20ds**_—ANOVA for the effect of **1 h Refed day** on the **cEdU index** and pairwise comparisons between days (Tukey’s HSD) —**mOr2-Piwi1 cells**. **S2J Table. DAY 1**—ANOVA for the effect of **feeding regime** on the **cEdU index** and pairwise comparisons between regimes (Tukey’s HSD) in **mOr2-Piwi1 cells**. **S2K Table. DAY 5**—ANOVA for the effect of **feeding regime** on the **cEdU index** and pairwise comparisons between regimes (Tukey’s HSD) in **mOr2-Piwi1 cells**. **S2L Table. DAY 7**—ANOVA for the effect of **feeding regime** on the **cEdU index** and pairwise comparisons between regimes (Tukey’s HSD) in **mOr2-Piwi1 cells**
(XLSX)

S3 Table Details of statistical analyses applied related to S3 Fig. **S3A Table.** Flow cytometer analysis after continuous of EdU pulse during starvation—cell cycle phases defined as per DNA content in All cells. **S3B Table. T**_**5ds**_—ANOVA for the effect of **starvation day** on the **cEdU index** and pairwise comparisons between days (Tukey’s HSD)—**All cells. S3C Table. T**_**20ds**_—ANOVA for the effect of **starvation day** on the **cEdU index** and pairwise comparisons between days (Tukey’s HSD)—**ll cells. S3D Table.** Flow cytometer analysis after continuous of EdU pulse during *ad libitum*—cell cycle phases defined as per DNA content in All cells. **S3E Table. *T***_**5ds**_—ANOVA for the effect of ***ad libitum* day** on the **cEdU index** and pairwise comparisons between days (Tukey’s HSD)—**All cells. S3F Table. *T***_**20ds**_—ANOVA for the effect of ***ad libitum* day** on the **cEdU index** and pairwise comparisons between days (Tukey’s HSD)—**All cells. S3G Table.** Flow cytometer analysis after continuous of EdU pulse after 1 h Refed—cell cycle phases defined as per DNA content in All cells. **S3H Table. T**_**5ds**_—ANOVA for the effect of **1 h Refed day** on the **cEdU index** and pairwise comparisons between days (Tukey’s HSD)—**All cells. S3I Table. T**_**20ds**_—ANOVA for the effect of **1 h Refed day** on the **cEdU index** and pairwise comparisons between days (Tukey’s HSD)—**All cells. S3J Table. DAY 1**—ANOVA for the effect of **feeding and starvation day** on the **cEdU index** and pairwise comparisons between days (Tukey’s HSD) in **All cells. S3K Table. DAY 5**—**ANOVA for the effect of feeding and starvation day** on the **cEdU index** and pairwise comparisons between days (Tukey’s HSD) in **All cells. S3L Table. DAY 7**—**ANOVA for the effect of feeding and starvation day** on the **cEdU index** and pairwise comparisons between days (Tukey’s HSD) in **All cells.**
(XLSX)

S4 Table Details of statistical analyses applied related to Figs 3, S3, and S4. **S4A Table. Linear regression** analysis of EdU+ cell accumulation. **S4B Table. Growth model analysis** of EdU+ cell accumulation. **S4C Table. Gompertz growth** model analysis of EdU+ cell accumulation: **K** and **t50**
(XLSX)

S5 Table Details of statistical analyses applied related to Figs 4 and S5. **S5A Table.** Flow cytometer analysis after 30 min of EdU pulse—cell cycle phases defined as per DNA content—pH3 index. **S5B Table.** T_5ds_/T_20ds_—ANOVA for the effect of **starvation day** on the fraction of **S-phase cells, G2/M-phase cells EdU index and pH3 index** and pairwise comparisons between **T**_**5ds**_ **and T**_**5ds**_ **+9 h/T**_**20ds**_ **and T**_**20ds**_ **+9 h** (Tukey’s HSD). **S5C Table. T**_**5ds**_—ANOVA for the effect of **feeding and starvation day** on the **EdU index** and pairwise comparisons between days (Tukey’s HSD). **S5D Table. T**_**5ds**_—ANOVA for the effect of **feeding and starvation day** on the fraction of **S-phase cells** and pairwise comparisons between days (Tukey’s HSD). **S5E Table. T**_**5ds**_—ANOVA for the effect of **feeding and starvation day** on the **pH3 index** and pairwise comparisons between days (Tukey’s HSD). **S5F Table. T**_**20ds**_—ANOVA for the effect of **feeding and starvation day** on the **EdU index** and pairwise comparisons between days (Tukey’s HSD). **S5G Table. T**_**20ds**_—ANOVA for the effect of **feeding and starvation day** on the fraction of **S-phase cells** and pairwise comparisons between days (Tukey’s HSD). **S5H Table. T**_**20ds**_—ANOVA for the effect of **feeding and starvation day** on the **pH3 index** and pairwise comparisons between days (Tukey’s HSD)
(XLSX)

S6 Table Details of statistical analyses applied related to Figs 4 and S6. **S6A Table**. Flow cytometer analysis—median fluorescent intensity H3K27ac in mOr2-Piwi1 cells. **S6B Table.** ANOVA for the effect of **feeding scenario** on the MFI **H3K27ac**—**mOr2-Piwi1 cells. S6C Table.** Flow cytometer analysis—median fluorescent intensity H3K27me3 in mOr2-Piwi1 cells. **S6D Table.** ANOVA for the effect of **feeding scenario** on the MFI **H3K27me3**—**mOr2-Piwi1 cells. S6E Table.** Flow cytometer analysis—median fluorescent intensity H3K27ac in All cells. **S6F Table.** ANOVA for the effect of **feeding scenario** on the MFI **H3K27ac**—**All cells. S6G Table.** Flow cytometer analysis—median fluorescent intensity H3K27me3 in All cells. **S6H Table.** ANOVA for the effect of **feeding scenario** on the MFI **H3K27me3**—**All cells.**
(XLSX)

S7 Table Details of statistical analyses applied related to Figs 5, S9 and S10. **S7A Table.** Flow cytometer analysis after 30 min of EdU pulse—cell cycle phases defined as per DNA content—pRPS6 index. **S7B Table.** T_5ds_/T_20ds_—ANOVA for the effect of **starvation day** on the fraction of **S-phase cells, EdU index and pRPS6 index** and pairwise comparisons between **T**_**5ds**_ **and T**_**5ds**_ **+9 h/T**_**20ds**_ **and T**_**20ds**_ **+9 h** (Tukey’s HSD). **S7C Table.** Flow cytometer analysis after 30 min of EdU pulse—pRPS6 index. **S7D Table. T**_**5ds**_—**Pearson correlation** between EdU+ and pRPS6+ cells. **S7E Table. T**_**20ds**_—**Pearson correlation** between EdU+ and pRPS6+ cells. **S7F Table. T**_**20ds**_ **to 30hpf**—**Pearson correlation** between EdU+ and pRPS6+ cells. **S7G Table. 33hpf to 72hpf**—**Pearson correlation** between EdU+ and pRPS6+  cells. **S7H Table.** Flow cytometer analysis cell cycle phases defined as per DNA content, of pRPS6+ and pRPS6- cells. **S7I Table.** Log2FC pRPS6+/pRPS6− Cell cycle phases.
(XLSX)

S8 Table Details of statistical analyses applied related to Figs 5 and S11. **S8A Table.** Western blot analysis after Rapamycin and AZD-8055 treatments—pRPS6/Actin levels. **S8B Table.** Student *t* test pairwise comparisons between relative **pRPS6/Actin levels** from **Rapamycin-**treated samples and controls. **S8C Table.** Student *t* test pairwise comparisons between relative **pRPS6/Actin levels** from **AZD-8055-**treated samples and controls. **S8D Table.** Rapamycin treatment—Flow cytometer analysis after 30 min of EdU pulse—cell cycle phases defined as per DNA content. **S8E Table. T**_**5ds**_**/T**_**20ds**_—Student *t* test pairwise comparisons between the **EdU index** from **Rapamycin**-treated samples and controls with **short EdU pulse. S8F Table. T**_**5ds**_**/T**_**20ds**_—Student *t* test pairwise comparisons between the fraction of **S-phase cells** from **Rapamycin**-treated samples and controls with **short EdU pulse. S8G Table.** AZD-8055 treatment—Flow cytometer analysis after 30 min of EdU pulse—cell cycle phases defined as per DNA content. **S8H Table. T**_**5ds**_**/T**_**20ds**_—Student *t* test pairwise comparisons between the **EdU index** from **AZD-8055** treated samples and controls with **short EdU pulse. S8I Table. T**_**5ds**_**/T**_**20ds**_—Student *t* test pairwise comparisons between the fraction of **S-phase cells** from **AZD-8055**-treated samples and controls with **short EdU pulse. S8J Table.** Rapamycin treatment—Flow cytometer analysis after continuous EdU pulse after 1 h Refed. **S8K Table. T**_**5ds**_**/T**_**20ds**_—Student *t* test pairwise comparisons between the **cEdU index** from **Rapamycin**-treated samples and controls with **continuous EdU pulse** after **1 h Refed. S8L Table.** Rapamycin treatment—Flow cytometer analysis after continuous EdU pulse during starvation. **S8M Table. T**_**5ds**_**/T**_**20ds**_—Student *t* test pairwise comparisons between the **cEdU index** from **Rapamycin**-treated samples and controls with **continuous EdU pulse** during **starvation.**
(XLSX)

S1 Data File containing individual numerical values for experiments depicted in the main and supplementary figures. (XLSX)

S1 Raw Images Raw images of Western blots corresponding to the cropped images in S11 Figs. Western blots show protein levels of pRPS6 and of Actin as loading control after refeeding and incubation for 1 day **(A, C, E, G)** or 2 days **(B, D, F, H)** with 0.2% DMSO, 4 µM or 20 µM Rapamycin (‘Rap’, A–D), or 0.1 µM or 1 µM AZD-8055 (‘AZD’, E–H). For each blot, the approximate protein size was determined using a protein ladder (‘PL’). ‘X’ in A, C, E, and G indicates three replicates of control protein extracts.
(PDF)

## Abbreviations

AL: ad libitum
cEdU: continuous EdU incubation
cEdU index: proportion of cell labelled by continuous EdU
DMSO: dimethyl sulfoxide
EdU: 5-ethynyl-2′-deoxyuridine
EdU index: proportion of cell labelled by EdU pulse
FC: flow cytometry
H3K27ac: acetylation of lysine 27 on histone H3
H3K27me3: trimethylation of lysine 27 on histone H3
MFI: median fluorescence intensity
pH3: phosphorylated histone H3 (mitotic marker)
pRPS6: phosphorylation of the ribosomal protein S6
PTMs: post-translational modifications
Rap: Rapamycin
T5ds: 5 days starvation
T20ds: 20 days starvation
TOR: target of Rapamycin
