# Supplementary figures and images for: Prolonged starvation deepens quiescence in Vasa2/Piwi1-expressing cells of a sea anemone
Source: PLoS Biol. 2025 Dec 8;23(12):e3003525. doi: 10.1371/journal.pbio.3003525 (PMC12704899; doi:10.1371/journal.pbio.3003525)

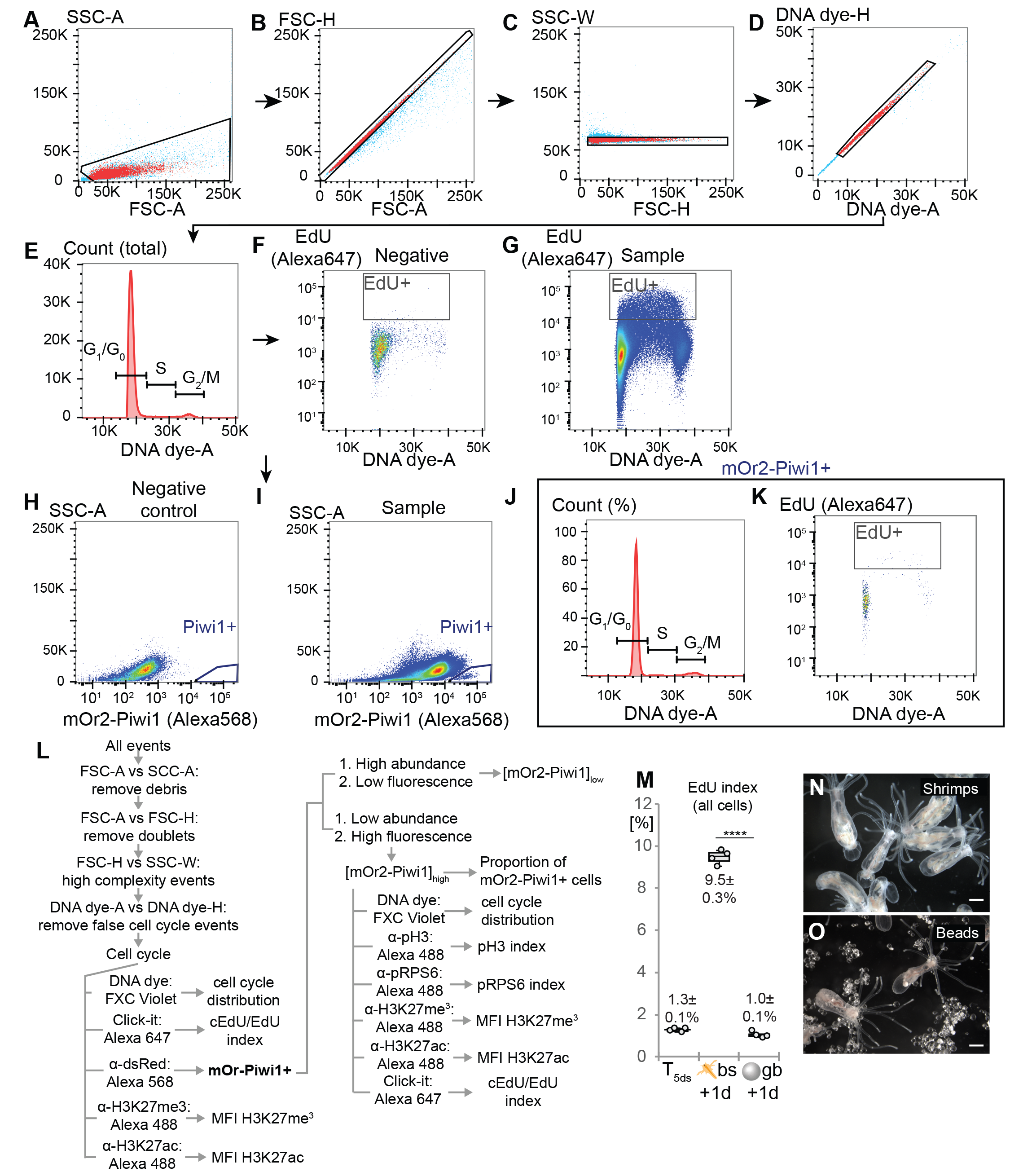

Supplement: S1 Fig — (A–C) Debris was excluded based on size and granularity in the FSC-A/SSC-A gate (A), with sub-gates based on FSC-A/FSC-H (B) and FSC-A/SSC-W (C) to remove potential cell doublets and high-complexity events. (D, E) Then particles were gated based on DNA dye intensity in width-over-area plots, and a histogram of DNA dye intensity (area, linear scale) was created to visualize characteristic DNA peaks corresponding to cells between 2N and 4N. (F, G) A threshold for EdU+ cells was determined based on the fluorescence signal of DMSO controls within the 2N–4N pool. Cells above this threshold were considered as EdU+. (H, I) Similarly, a threshold for mOr2-Piwi1+ cells was drawn based on the fluorescence signal of negative controls (no primary antibody) within the 2N–4N pool, identifying small and bright cells as mOr2-Piwi1+. (J, K) Predefined cell cycle phases and EdU+ cells were then analyzed within this pool of cells. (L) Hierarchical logic used to define cell cycle phases, followed by quantification of mOr2-Piwi1+ cells and calculation of the EdU, cEdU, pH3, and pRPS6 indices, and the median fluorescent intensity of H3K27ac and H3K27me3 within mOr2-Piwi1+ cells. The parameters used for gating and analysis are specified at each step. (M, N, O) Incubation with brine shrimps (‘bs’; M, N), but not BSA-coated glass beads (‘gb’; M, O) induces S-phase re-entry 24 hours after incubation at T5ds. Representative stereomicroscopy images of juvenile polyps fed with brine shrimps (N) or BSA-coated glass beads (O) after approx. 1 h of incubation. Note that glass beads were taken up and expanded the body cavity similarly to brine shrimps. Scale bar: 0.5 mm. See Data visualization for definition of box plots and bar plots. Values in M represent means ± standard deviations with dots representing individual samples. n = 4 biological replicates per condition, each replicate consisting of a pool of 15 animals. Significance levels after one-way ANOVA with Tukey’s HSD for pairwise comparisons [file pbio.3003525.s001.tif]

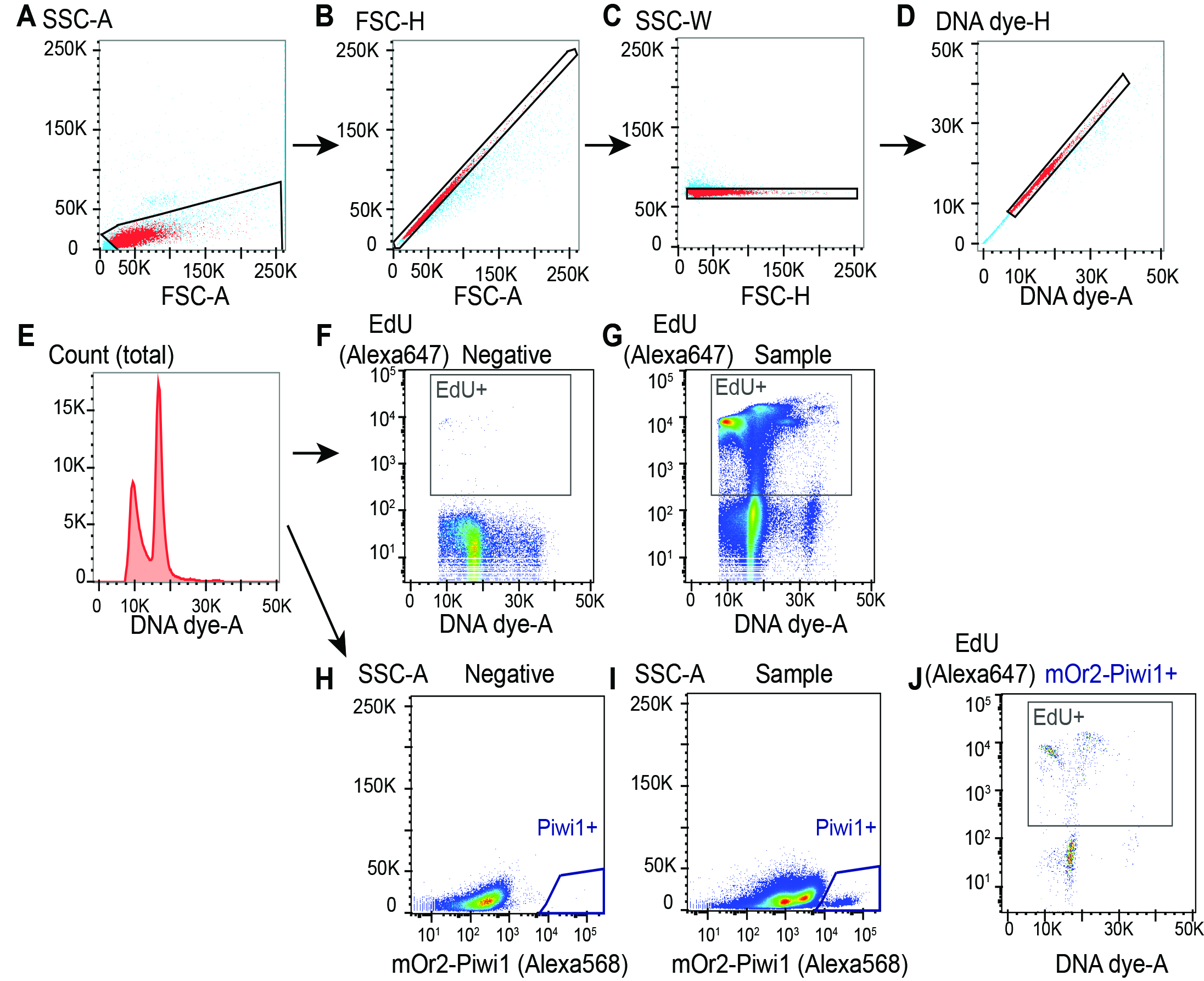

Supplement: S2 Fig — (A–C) Debris was excluded based on size and granularity in the FSCA-/SSC-A gate (A), with sub-gates based in FSC-A/FSC-H (B), and FSC-A/SSC-W (C) to remove potential cell doublets and high-complexity events. (D, E) Then, particles were gated based on DNA dye intensity in width-over-area plots, and a histogram of DNA dye intensity (area, linear scale) was created to visualize characteristic peaks corresponding to cells between 2N and 4N. We observed that long-term incorporation of EdU interfered with the DNA stain fluorescence and prevented a clear identification of 2N–4N cells. Therefore, we used a broader range of DNA intensity to define the parental gate of EdU+ populations. (F, G) A threshold for EdU+ cells was determined based on the fluorescence signal of DMSO controls within the pool. (H, I) Similarly, a threshold for mOr2-Piwi1+ cells was drawn based on the fluorescence signal of negative controls (no primary antibody), identifying small and bright cells as mOr2-Piwi1+. (J) The same gates were applied to analyze cell cycle composition and the fraction of EdU+ cells within this pool of cells. (TIF) [file pbio.3003525.s002.tif]

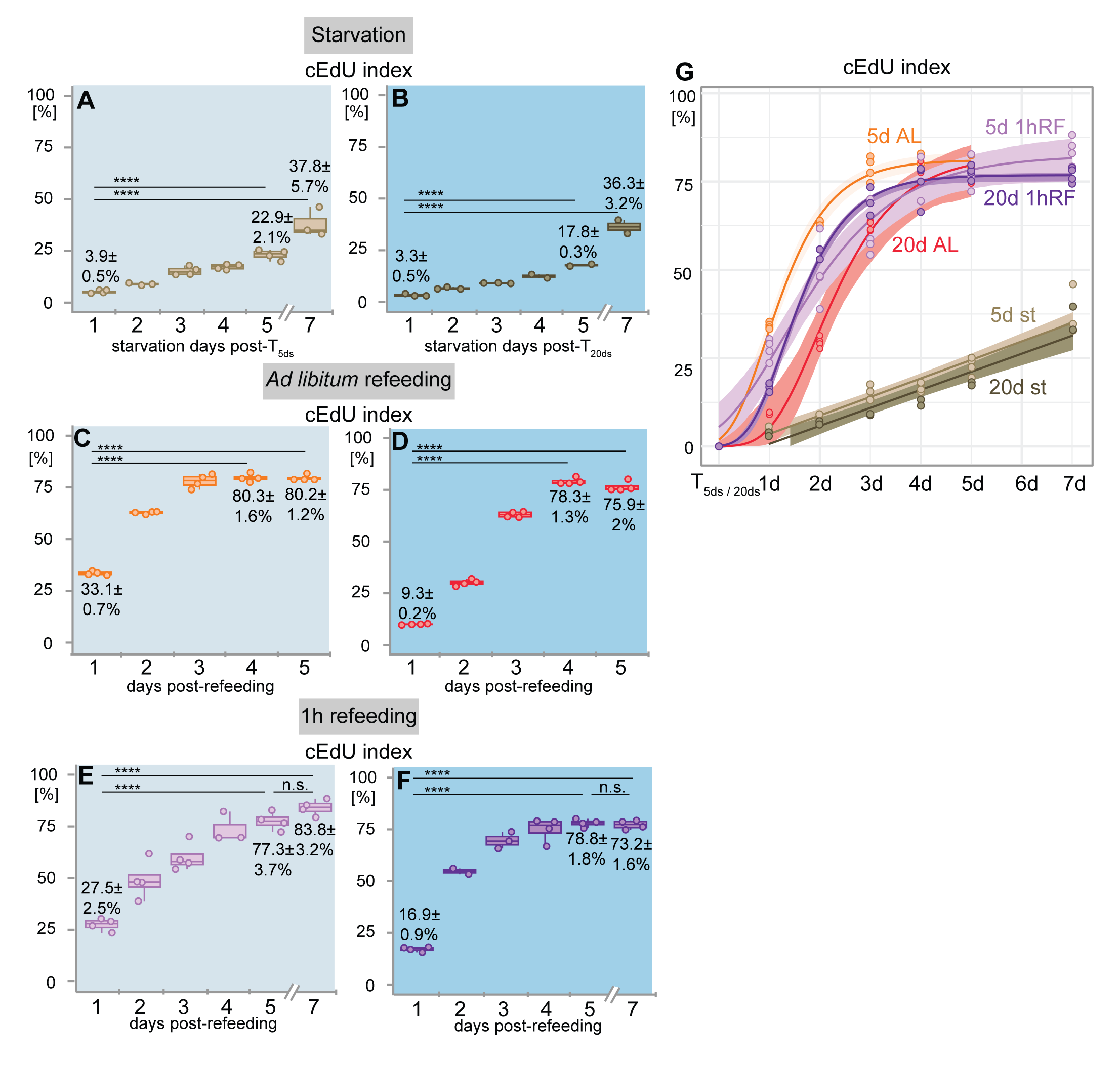

Supplement: S3 Fig — (A–F) Temporal changes among all cells in the cumulative EdU (cEdU) index under continued starvation (A, B), ad libitum refeeding (C, D), or following a single, 1-hour refeeding pulse (E, F) after 5 (T5ds, A, C, E) or 20 days (T20ds, B, D, F) of starvation. See Fig 1 for schematic of experimental setups. Experiments were done using flow cytometry. (G) Dynamics of the cEdU index (A–F) are best explained by linear growth models under continued starvation (st), or by Gompertz growth models after ad libitum (AL) or a 1-hour refeeding pulse (1hRF). Dots represent same replicate sample values as in (A–F). n = 2–4 biological replicates per condition (15 individuals per replicate). Coloured lines in G represent the model curve or line for each condition with overlays depicting 95% confidence intervals. See Data visualization for definition of box plots. Dots represent individual values. Index values represent means ± standard deviations of respective timepoints. Pairwise comparisons after one-way ANOVA were calculated using Tukey’s HSD and p values adjusted at significance codes: ****p < 0.0001. d: day(s), n.s.: non-significant. See S3 and S4 Tables for mean values and statistical data and S1 Data for individual numerical values. (TIF) [file pbio.3003525.s003.tif]

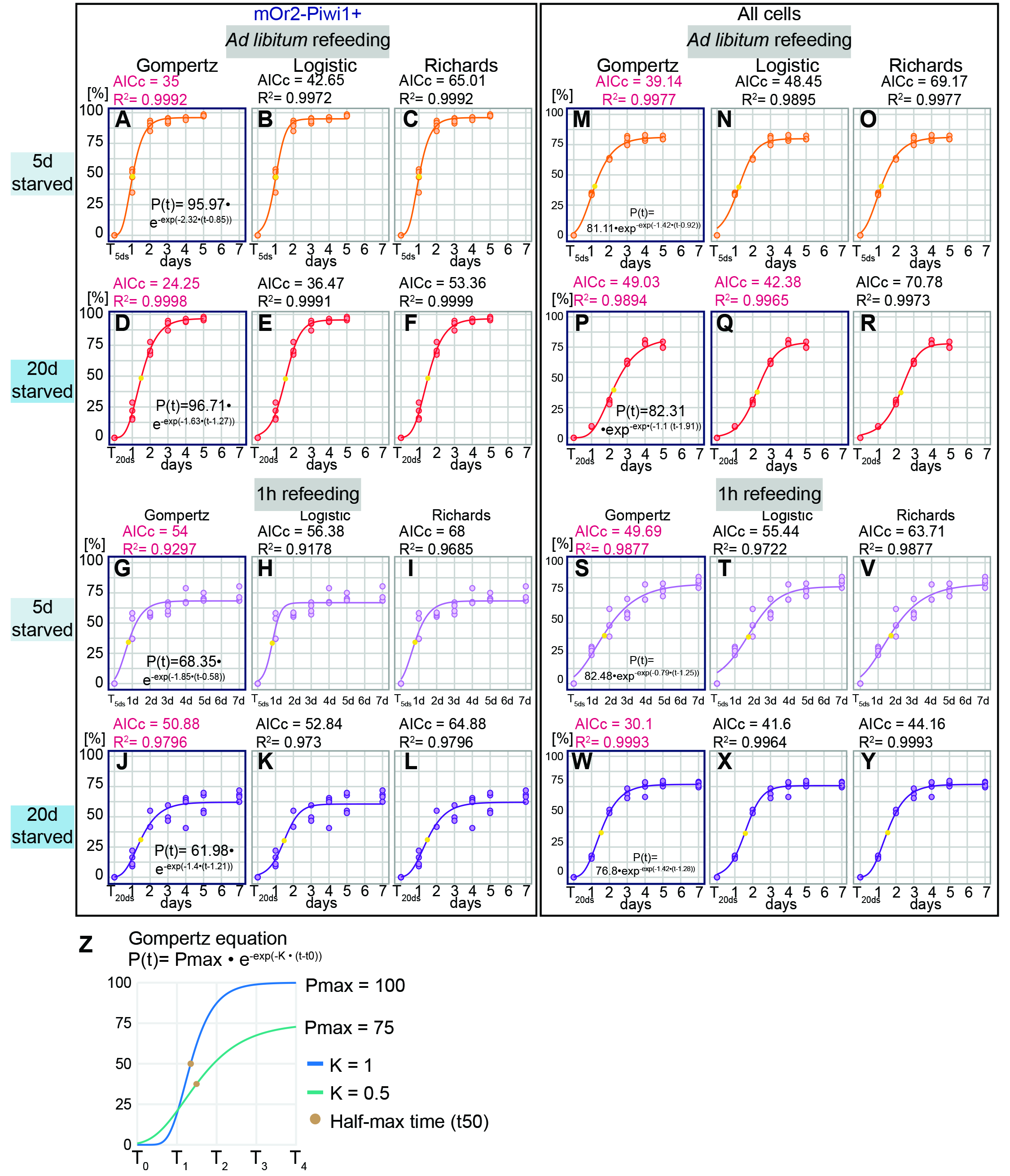

Supplement: S4 Fig — (A–Y) Comparison of Gompertz (A, D, G, J, M, P, S, W), Logistic (B, E, H, K, N, Q, T, X), and Richards (C, F, I, L, O, R, V, Y) growth models for mOr2-Piwi1+ (A–L) and all cell cycle-gated cells (M–Y) during ad libitum (A–F, M–R) and 1-hour refeeding conditions (G–L, S–Y). The best fitting model for each condition was chosen based on the highest coefficient of determination (R2) and lowest Akaike Information Criterion corrected (AICc). Overall conditions, the Gompertz growth model performed best (red values). n = 2–4 biological replicates per condition (15 individuals per replicate). Black lines represent the growth model curve for each condition. Dots represent individual values. The Gompertz equation is displayed. (Z) The Gompertz growth model assumes a maximum value and exponential decay as the population approaches this maximum. The growth rate constant (K) controls how quickly the curve transitions and indicates the speed at which the predicted maximum (Pmax) is reached. The half-max time (t50) indicates the time point where the function reaches 50% of the Pmax. d: day(s). See S4 Table for mean values and statistical data and S1 Data for individual numerical values. (TIF) [file pbio.3003525.s004.tif]

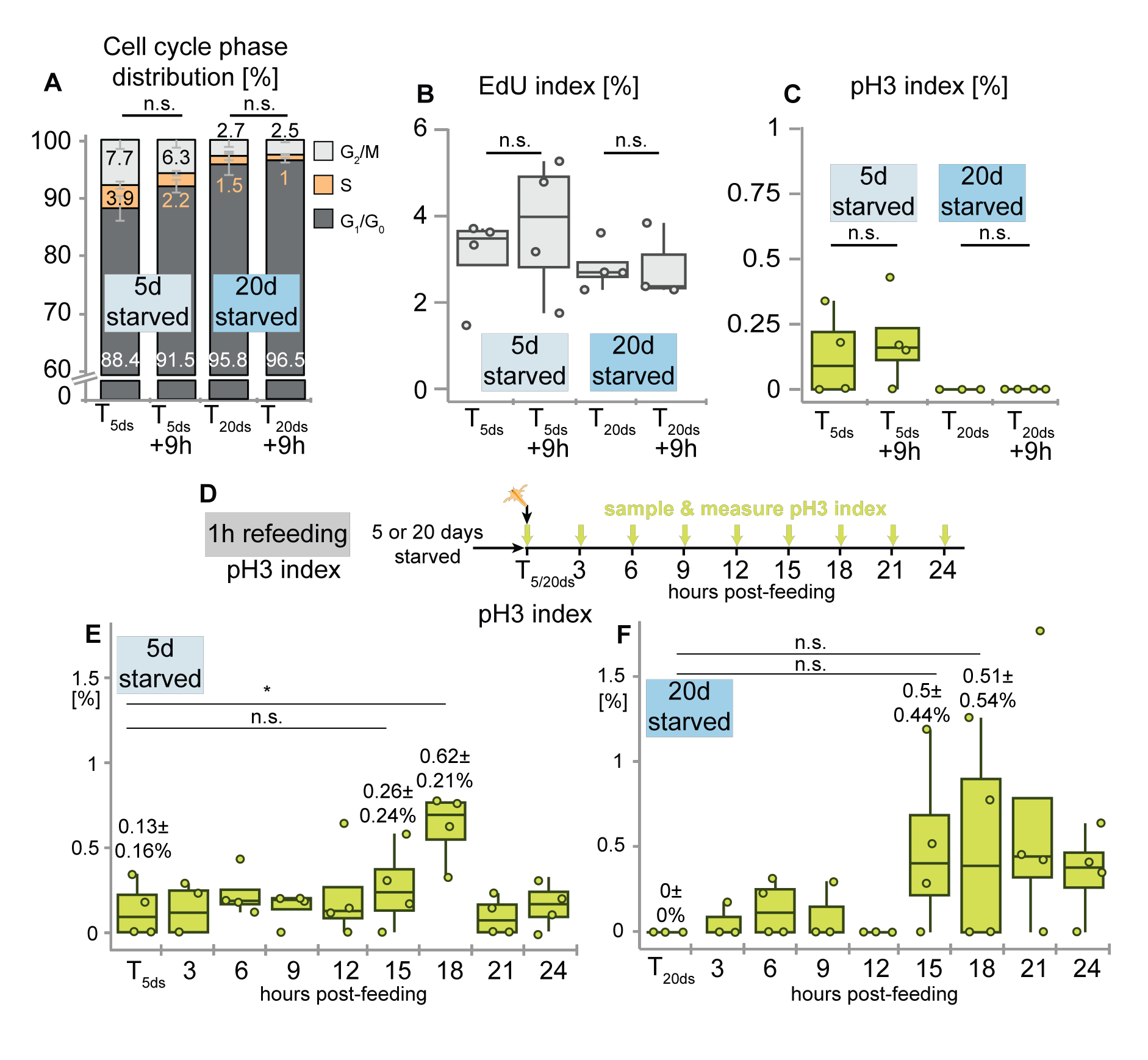

Supplement: S5 Fig — (A–C) The cell cycle phase distribution (A), proportion of EdU+ cells (EdU index; B), and proportion of pH3+ cells (pH3 index; C) of Vasa2+/Piwi1+ cells from 5 or 20 days starved polyps (T5ds or T20ds) sampled 9 hours apart showed no significant difference. (D) Schematic illustrating the sampling, feeding regimes, and EdU incubations. Polyps starved for 5 or 20 days (T5ds or T20ds) were refed once for 1 hour and sampled at indicated hours post-refeeding. (E, F) Quantification of the pH3 index over 24 hours after refeeding in polyps starved for 5 or 20 days showed no significant difference between T5ds or T20ds and 15-h post-refeeding regardless of the starvation length. All experiments were done using flow cytometry. For box plots and bar plot definitions, see Data visualization. Dots in (B, C, F, G) represent individual values. Values in (A, E, F) represent means ± standard deviations at respective timepoints with dots representing individual samples. n = 3–4 biological replicates per condition, with 15 polyps per replicate. Pairwise comparisons after one-way ANOVA were calculated using Tukey’s HSD and p values adjusted at significance codes: *p < 0.05. n.s.: non-significant. See S5 Table for mean values and statistical data and S1 Data for individual numerical values. (TIF) [file pbio.3003525.s005.tif]

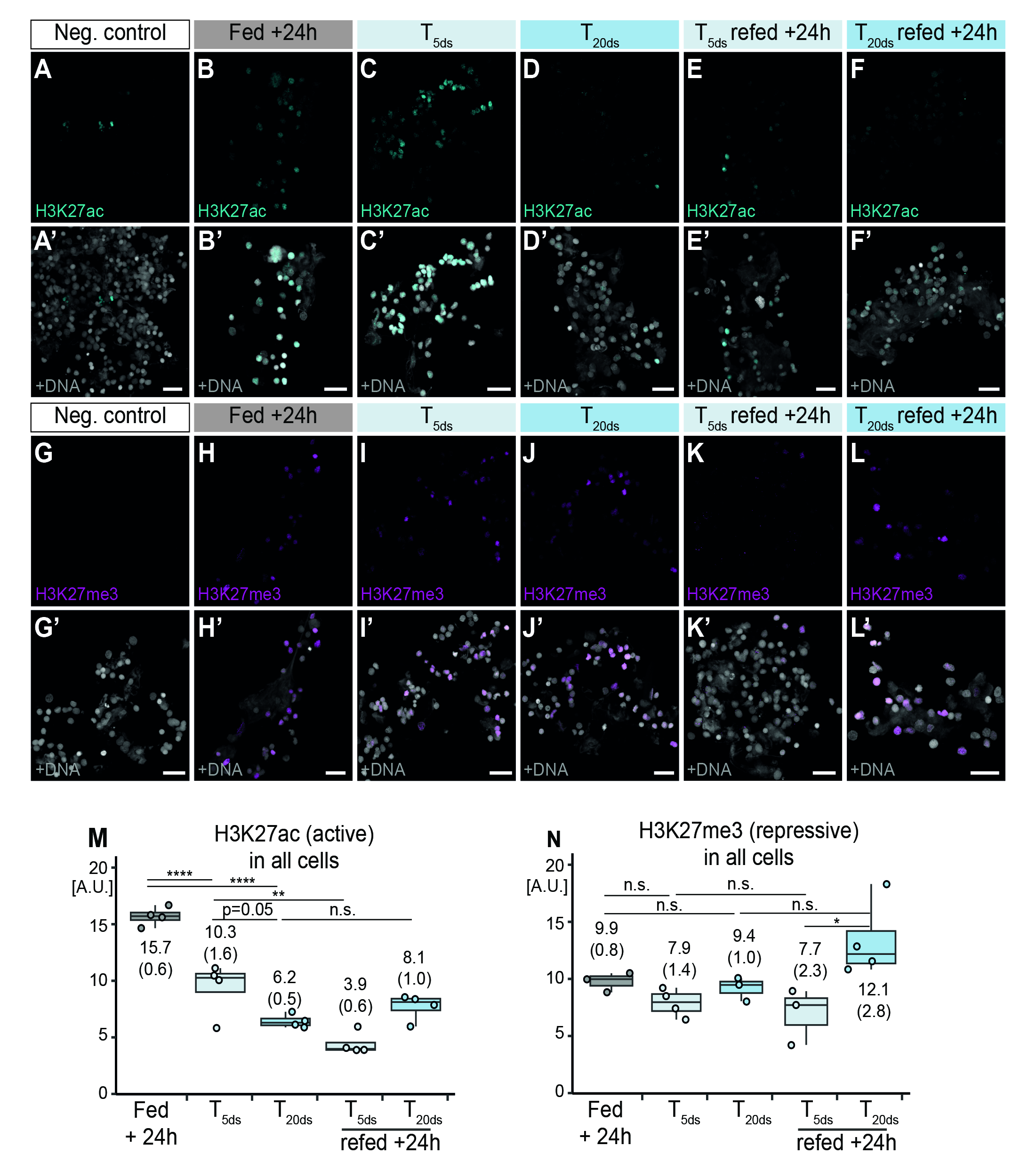

Supplement: S6 Fig — (A–L′) Projections of confocal imaging stacks of cell clumps dissociated from juvenile polyps and immunolabeled against H3K27ac (A–F′) and H3K27me3 epitopes (G–L′). Both histone marks colocalized with nuclei labeled by Hoechst33342 DNA dye (A′–L′). Scale bar in A′–L′: 10 µm. (M, N) Comparison of the median fluorescent intensity (MFI) of H3K27ac (M) and H3K27me3 (N) at 24 h after continuous feeding (Fed + 24 h), at starvation (T5ds, T20ds) or at 24-h post-refeeding (T5ds/T20ds refed + 24 h). Between fed, T5ds and T20ds timepoints, MFI levels of H3K27ac progressively and significantly decreased while levels H3K27me3 (M) did not change significantly (N). Starved polyps were refed for 1 hour. For box plots and bar plot definitions, see Data visualization. Values in M and N represent median and interquartile range (IQR) of respective timepoints with dots indicating individual samples. n = 2–4 biological replicates per condition, with 15 polyps per replicate. Significance levels after one-way ANOVA with Tukey’s HSD for pairwise comparisons are indicated for adjusted p values: *p < 0.05, **p < 0.01; ****p < 0.0001. d: day(s), n.s.: non-significant. See S6 Table for mean values and statistical data and S1 Data for individual numerical values. (TIF) [file pbio.3003525.s006.tif]

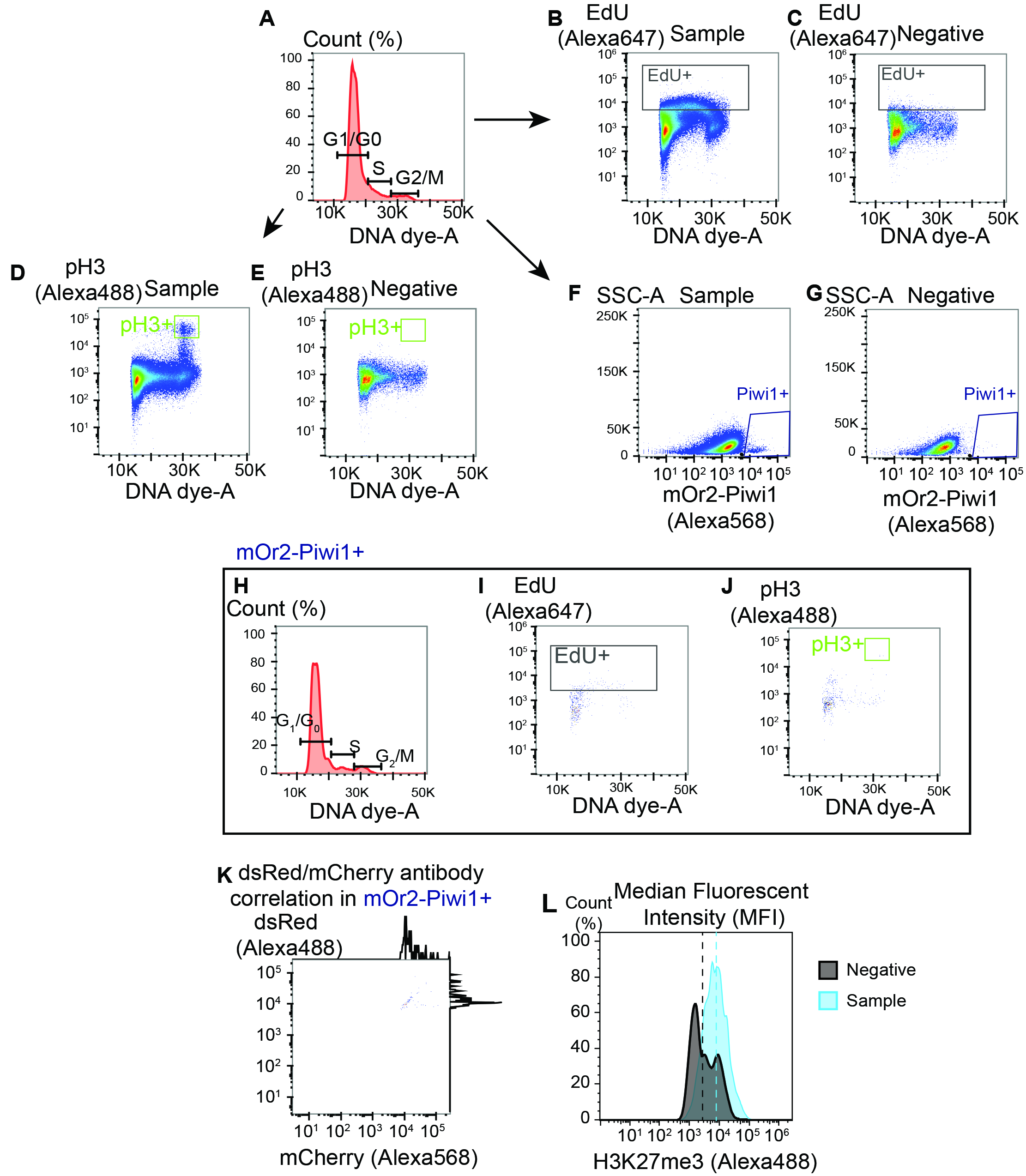

Supplement: S7 Fig — (A) Debris was excluded, and cells were gated based on DNA dye intensity as described above. (B, C) For EdU+ cells, a threshold was drawn above the fluorescence signal of DMSO controls within the 2N–4N pool. (D, E) For pH3+ cells, a threshold was drawn based on the fluorescence signal of negative controls (no primary antibody) within the 2N–4N pool, identifying G2/M-phase cells as expected. (F, G) Similarly, for mOr2-Piwi1+ cells, a threshold was drawn based on the fluorescence signal of negative controls within the 2N–4N pool, identifying a small population of bright cells. (H–J) The same gates were applied to analyze cell cycle phases and the proportion of pH3+ and EdU+ cells within this pool of cells. (K) Comparison between the use of mCherry and dsRed antibodies for immunolabeling mOr2-Piwi1 cells. mOr2-Piwi1 was detected using both an mCherry antibody coupled with Alexa568 and a dsRed antibody coupled with Alexa488. Debris was excluded, and cells were gated based on DNA-dye intensity as explained above. For mOr2-Piwi1+ cells, thresholds were drawn based on the fluorescence signal of negative controls (no primary antibody) within the 2N–4N pool. A linear correlation between the fluorescent signals was confirmed. (L) Histogram comparing the fluorescent intensity distribution of a negative control and a sample stained for histone markers H3K27ac or H3K27me3. The median fluorescent intensity (MFI) for each population is indicated by a dashed line. (TIF) [file pbio.3003525.s007.tif]

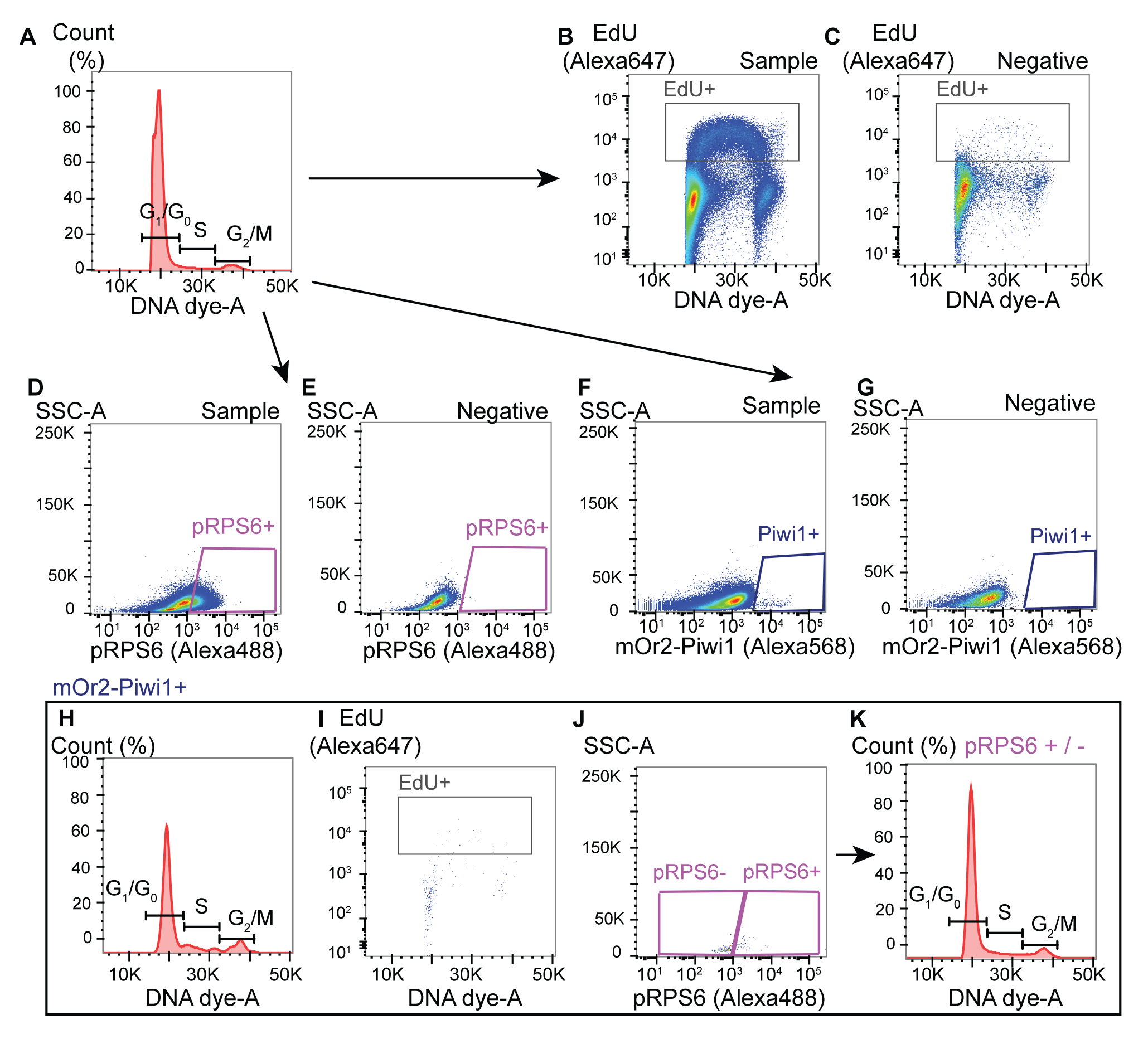

Supplement: S8 Fig — (A) Debris was excluded and gated cells based on DNA-dye intensity as explained above. (B, C) For analyzing EdU+ cells, a threshold was drawn above the fluorescence signal of DMSO controls within the 2N–4N pool of cells. (D, E) For pRPS6+ and pRPS6− cells, thresholds were drawn based on the fluorescence signal of negative controls (no primary antibody) within 2N–4N pools of cells. (F, G) For mOr2-Piwi1+ cells, a threshold was drawn based on the fluorescence signal of negative controls (no primary antibody) within 2N–4N pools of cells. (H–K) The same gates were applied to analyze cell cycle phases and the proportion of pRPS6+ and EdU+ cells within this pool of cells. (TIF) [file pbio.3003525.s008.tif]

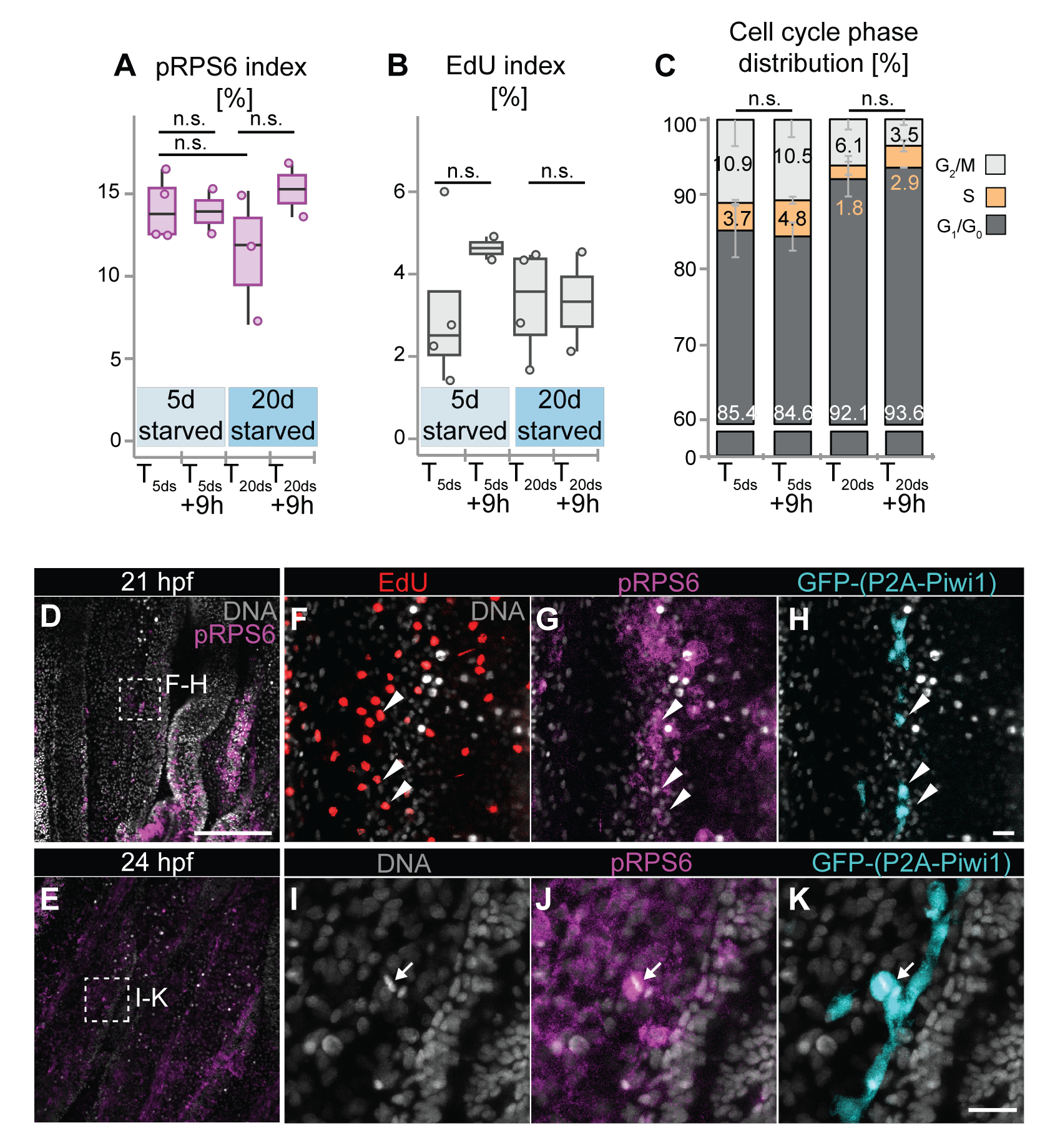

Supplement: S9 Fig — (A–C) The proportion of phospho-ribosomal protein S6-positive cells (pRPS6 index; A), EdU+ cells (EdU index; B), and cell cycle phase distribution (C) of Vasa2+/Piwi1+ cells sampled 9 h apart at T5ds or T20ds showed no significant differences. Experiments were done using flow cytometry. For box plots and bar plot definitions, see Data visualization. Dots in (A, B) represent individual values. Values in (C) represent means. n = 2–4 biological replicates per condition, with 15 polyps per replicate. (D–K) Confocal imaging stacks of gastrodermal tissue from Piwi1P2A-GFP juvenile polyps. (D, E) Overview of mesenteries at midbody level of whole-mount polyps stained by immunofluorescence against pRPS6 sampled at 21 h or 24 h post-refeeding (hpf) after 5 days of starvation. Side views with oral side oriented downwards. (F–H) Single cells co-labeled by EdU, pRPS6, and Piwi1-(P2A-GFP)(white arrowheads). (I–K) A single metaphase cell co-labeled by pRPS6 and Piwi1-(P2A-GFP)(white arrow). EdU pulse labeling started 30 min before fixation. Gray: Hoechst DNA dye. Scale bar: 100 µm (D, E) and 10 µm (F–K). n.s.: non-significant. See S7 Table for mean values and statistical data, S1 Data for individual numerical values. (TIF) [file pbio.3003525.s009.tif]

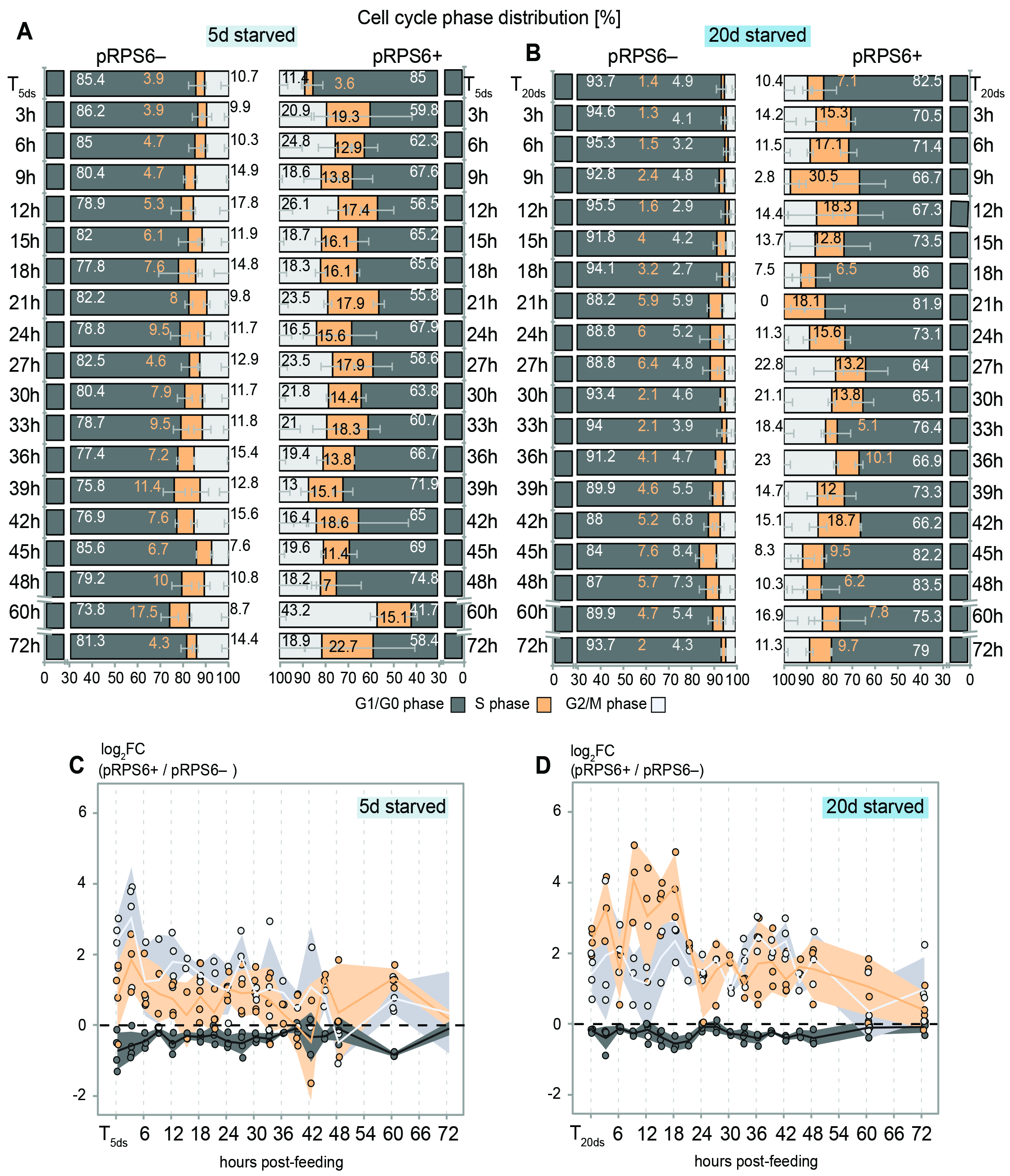

Supplement: S10 Fig — (A, B) Flow cytometry-based cell cycle phase distributions of pRPS6+ and pRPS6− cells over 72 hours after refeeding at T5ds (A) or T20ds (B). Polyps were refed for 1 hour and sampled at indicated time points. For bar plot definitions, see Data visualization. Values in (A, B) represent means. n = 2–4 biological replicates per condition, with 15 polyps per replicate. (C, D) Log2FC of the ratio of the cell cycle fractions between pRPS6+ and pRPS6− cells. Note that regardless of the starvation duration, the pRPS6+ cells are overrepresented (Log2FC > 0) in the S and G2/M fractions upon refeeding. Dots represent individual samples. Coloured lines indicate mean values for each cell cycle phase and band overlays represent 95% confidence intervals. n = 2–4 biological replicates per condition, with 15 polyps per replicate. See S7 Table for mean values and statistical data and S1 Data for individual numerical values. (TIF) [file pbio.3003525.s010.tif]

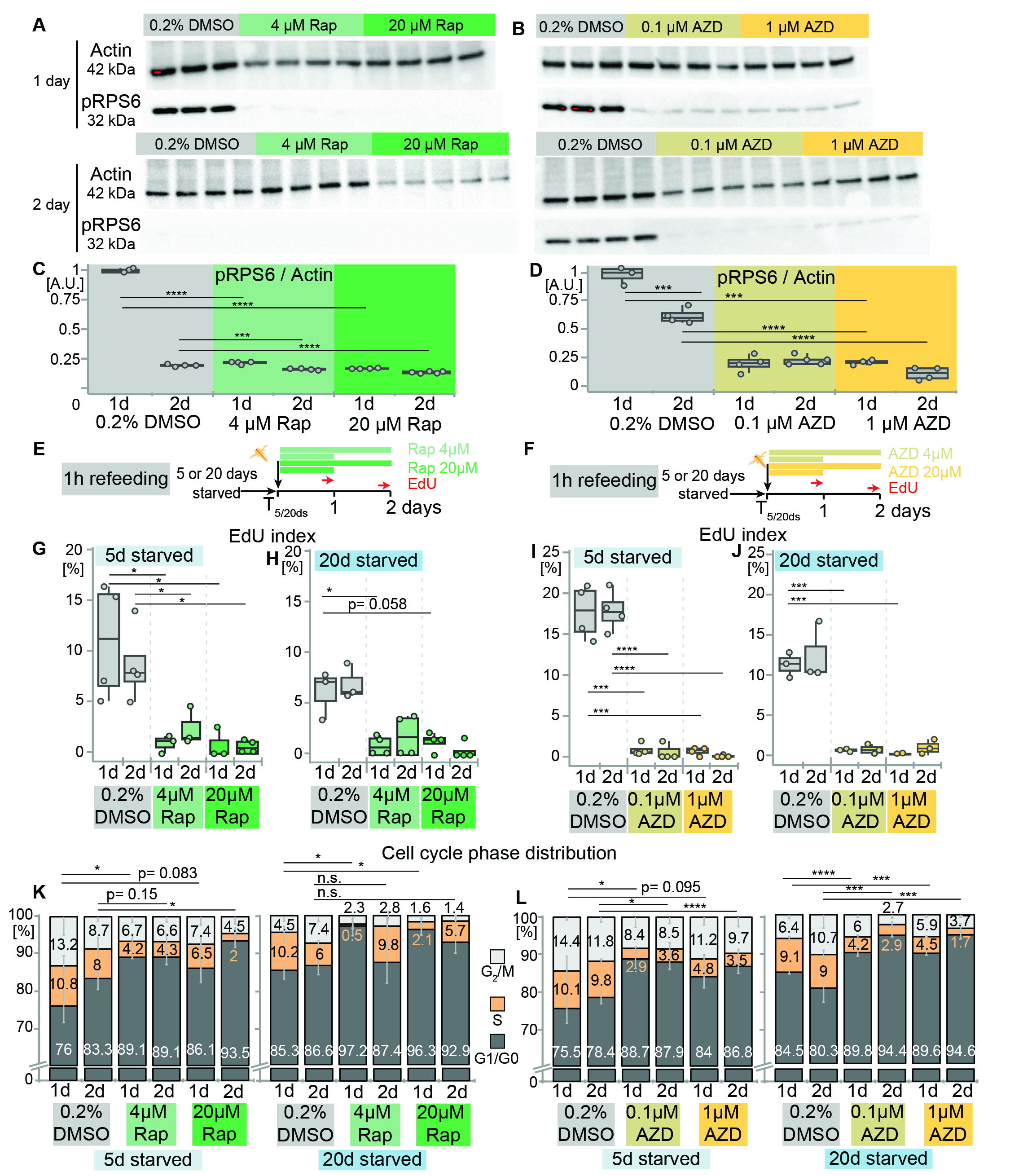

Supplement: S11 Fig — (A, B) Western blots depict protein levels of phosphorylated ribosomal protein S6 (pRPS6) Actin (as control) after refeeding and incubating for 1 or 2 days with 0.2% DMSO, 4 µM or 20µM Rapamycin (‘Rap’, A) or 0.1 µM or 1µM AZD-8055 (‘AZD’, B). (C, D) Intensity measures of pRPS6 bands relative to the Actin control protein show that Rapamycin (C) and AZD-8055 (D) led to a decrease of phosphorylated RPS6 levels. n = 3–5 technical replicates from one biological replicate with pools of 50 Rapamycin-, AZD-8055- or 0,2% DMSO-treated polyps. (E, F) Schematics illustrating the feeding procedure, incubation conditions of EdU and the TOR inhibitors Rapamycin (‘Rap’, E) or AZD-8055 (‘AZD’, F), and sampling timepoints. After 5 or 20 days of starvation (T5ds or T20ds), polyps were refed for 1 hour. (G–L) Effect of Rapamycin (G, H, K) and AZD-8055 (I, J, L) treatment on the proportion of EdU (EdU index, G–J) and cell cycle phase distribution (K, L) after a 1 h-feeding pulse at T5ds or T20ds. Compared to 0.2% DMSO-treated controls, Rap (G, H) and AZD (I, J) treatment leads to a reduced EdU index regardless of starvation history, concentration, or incubation time. Rap (K) and AZD (L) reduce the fractions of S- and G2/M-phase cells. For box plots and bar plot definitions, see Data visualization. Dots represent individual values. n = 2–4 biological replicates per condition, with 15 individuals per replicate. Significance levels for Student t test are indicated for adjusted p values: *p < 0.05, ***p < 0.001, ***p < 0.0001. d: day(s), n.s.: non-significant. See S8 Table for mean values and statistical data, S1 Data for individual numerical values and S1 Raw Images for raw images of Western Blots. (TIF) [file pbio.3003525.s011.tif]

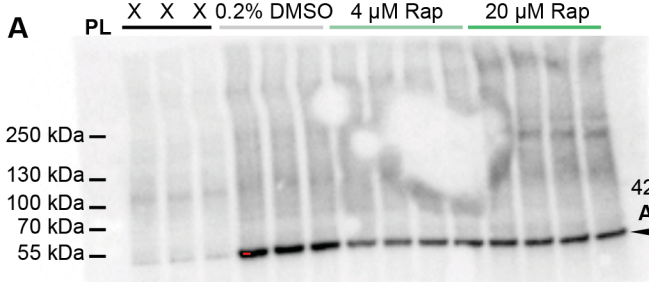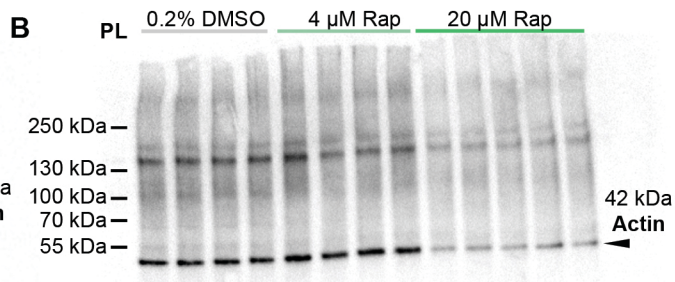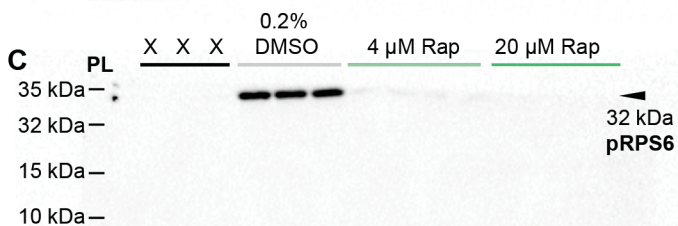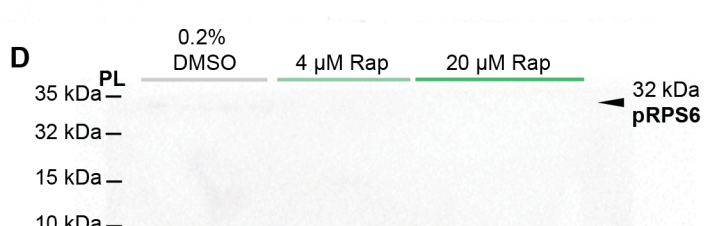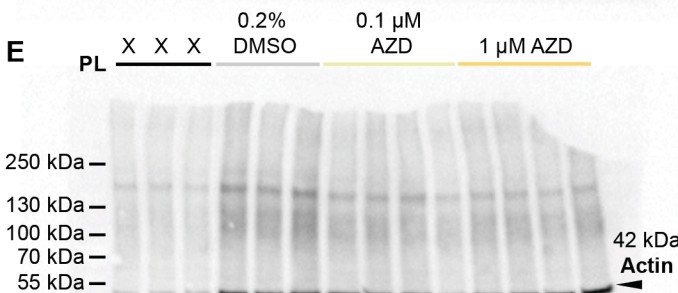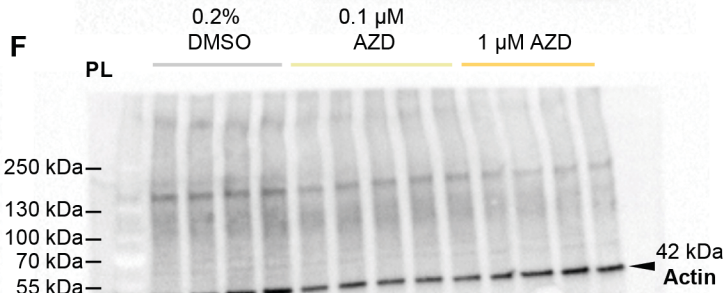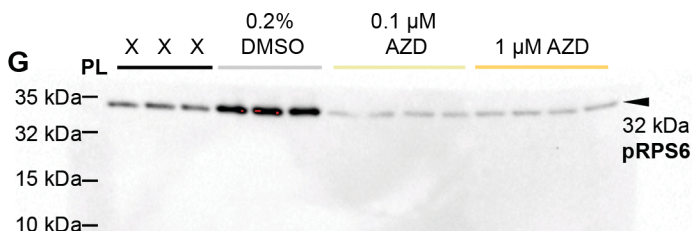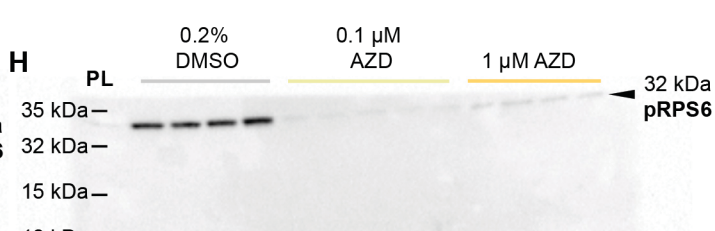

Supplement: S1 Raw Images — Western blots show protein levels of pRPS6 and of Actin as loading control after refeeding and incubation for 1 day (A, C, E, G) or 2 days (B, D, F, H) with 0.2% DMSO, 4 µM or 20 µM Rapamycin (‘Rap’, A–D), or 0.1 µM or 1 µM AZD-8055 (‘AZD’, E–H). For each blot, the approximate protein size was determined using a protein ladder (‘PL’). ‘X’ in A, C, E, and G indicates three replicates of control protein extracts. (PDF) [file pbio.3003525.s021.pdf]
